# New Advances in the Study of Regulation of Tomato Flowering-Related Genes Using Biotechnological Approaches

**DOI:** 10.3390/plants13030359

**Published:** 2024-01-25

**Authors:** Denis Baranov, Sergey Dolgov, Vadim Timerbaev

**Affiliations:** 1 Laboratory of Expression Systems and Plant Genome Modification, Branch of Shemyakin-Ovchinnikov Institute of Bioorganic Chemistry, Russian Academy of Sciences, 142290 Pushchino, Russia; d.y.baranov@yandex.ru (D.B.); dolgov@bibch.ru (S.D.); 2 Laboratory of Plant Genetic Engineering, All-Russia Research Institute of Agricultural Biotechnology, 127550 Moscow, Russia

**Keywords:** *Solanum lycopersicum* L., flower meristem development, fruit set, gene regulation, CRISPR/Cas9, RNA interference, gene silencing, gene overexpression

## Abstract

The tomato is a convenient object for studying reproductive processes, which has become a classic. Such complex processes as flowering and fruit setting require an understanding of the fundamental principles of molecular interaction, the structures of genes and proteins, the construction of signaling pathways for transcription regulation, including the synchronous actions of *cis*-regulatory elements (promoter and enhancer), trans-regulatory elements (transcription factors and regulatory RNAs), and transposable elements and epigenetic regulators (DNA methylation and acetylation, chromatin structure). Here, we discuss the current state of research on tomatoes (2017–2023) devoted to studying the function of genes that regulate flowering and signal regulation systems using genome-editing technologies, RNA interference gene silencing, and gene overexpression, including heterologous expression. Although the central candidate genes for these regulatory components have been identified, a complete picture of their relationship has yet to be formed. Therefore, this review summarizes the latest achievements related to studying the processes of flowering and fruit set. This work attempts to display the gene interaction scheme to better understand the events under consideration.

## 1. Introduction

Flowering plants have morphological diversity and can grow in different ecological niches. The transition of plants from the vegetative phase to the reproductive phase is a significant switch in their life cycle since the reproduction of offspring is the most essential function of all living things. The optimal timing of this event from a physiological point of view is a prerequisite for successful reproduction. Flowering and fruit set are initiated and regulated via the combined action of various genetic factors in response to endo- [1,2] and exogenous [3,4] stimuli. Processes, including male and female organogenesis, meiosis, gametogenesis, pollination, and fertilization, occur during the diploid and haploid phases of reproductive development, which are necessary to maintain genetic variability [5]. After successful pollination and fertilization of the ovary, the coordinated action of growth signals removes the inhibition of ovary development [6,7]. Concurrently, pistil senescence and flower abscission occur in the absence of positive stimuli. [8,9]. The functions of the flowering and development regulators are often conserved in different angiosperms; in this regard, tomato is a convenient object for studying the mechanisms of regulation at the gene level in climacteric fruits [4].

Tomato (*Solanum lycopersicum* L.) is a commercially important crop grown for fresh or processed consumption [10,11]. Self-compatibility and short life cycles (90–120 days) are appealing factors for agricultural producers [12], while high taste quality [13,14,15] and nutritional value [16,17,18] are important for consumers.

Genetic engineering methods have been used to study genetic factors regulating tomato reproduction for several decades [19]. A vast amount of knowledge has been accumulated, which gives an idea of the general picture of molecular interactions. However, creating new methodological tools makes it possible to significantly expand the knowledge concerning flowering, setting, and fruit development processes.

**Figure 1:**
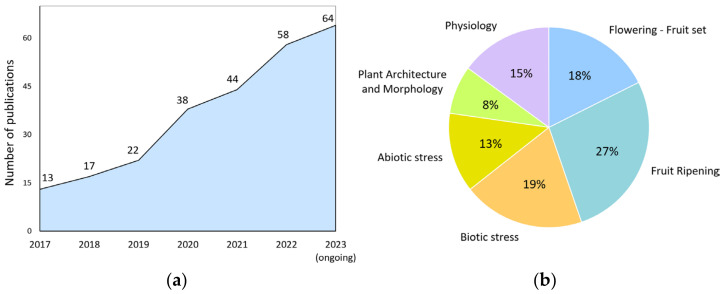
Publication activity of the world scientific community. (**a**) CRISPR/Cas9-related studies on tomato by year; (**b**) research topics focused on exploring various physiological processes in tomato (2017–2023) using CRISPR/Cas9 technology.

For example, precise genome editing by the CRISPR/Cas9 system allows researchers to create knockout alleles, make subtle changes to gene sequences, and even replace entire genes [20]. This, in turn, helps to study gene function, interactions, and regulation. Despite the 10-year history of using this powerful technology, the number of publications using it, in which tomato is the subject, is steadily increasing year by year (Figure 1a). Its research potential for scientists is far from being exhausted.

We analyzed the publications where the CRISPR/Cas9 system was applied on tomatoes for the last six years. This allowed us to identify the most exciting topics for the scientific community (Figure 1b). It turned out that almost half (45%) of the studies are devoted to the study of genes involved in tomato reproductive processes in general. Publications revealing narrower topics—flowering, fertilization, and fruit development—occupy 40% of this share (18% of the total publications). It turned out that many of the gene-regulatory genes encode transcription factors and co-regulators of transcription, miRNAs, or proteins involved in the epigenetic control of gene expression. The high research interest in this area is that, in many cases, these regulators’ molecular mechanisms of action still need to be fully understood. The stably relevant topic of stress (abiotic and biotic) occupies one-third of the total publications. The remaining publications cover fields devoted to other physiological processes (15%), plant architecture, and morphology (8%).

**Figure 2:**
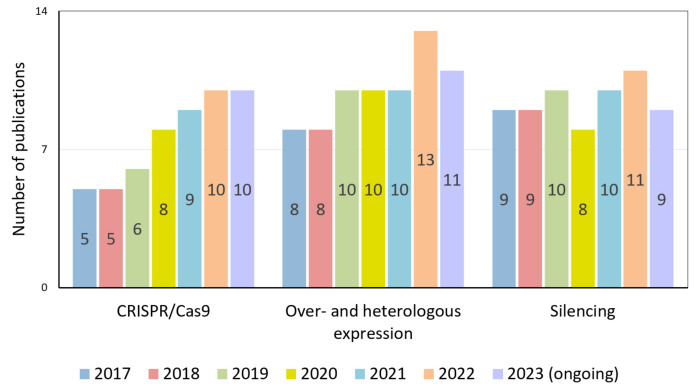
Number of publications by year devoted to studying tomato flowering processes using CRISPR/Cas9 technology, over- and heterologous expression approaches, and gene-silencing technologies.

Among the genetic engineering methods used to study the regulation of reproductive processes, the use of the CRISPR/Cas9 system is expected to increase (Figure 2).

At the same time, the approaches that have already become classical, such as the RNA interference (RNAi) silencing of gene expression and gene overexpression (OE), have not lost their relevance and the quantity of related publications has remained stably high over the last six years. Thus, despite the large chunk of accumulated scientific data, regulating reproductive processes still needs to be explained.

Thus, this review highlights the significant advances in the study of genes regulating tomato flowering obtained using directed genome-editing, silencing, and gene overexpression methods. Firstly, genes associated with the meristem transition from the vegetative to the reproductive phase are described. Then, the consideration of genes involved in flower formation is proposed, and, finally, genes involved in pollen maturation and fertility are outlined. In addition, based on the collected data, we propose a model of signaling regulation of tomato flowering.

## 2. Meristem Transition

Tomato shoot architecture is shaped by determinate and indeterminate meristems [21]. Indeterminate meristems are pools of undifferentiated plant cells capable of unlimited division and growth. In contrast, determinate meristems represent finite structures. Determinate reproductive meristems do not form de novo but originate from indeterminate meristems [22].

**Figure 3:**
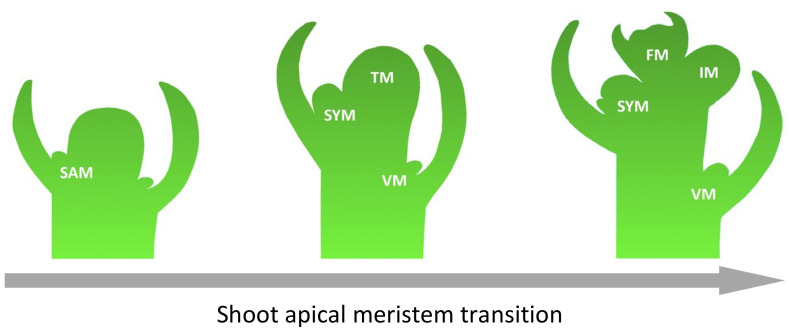
Steps of inflorescence formation in tomato. Abbreviations: shoot apical meristem (SAM), transitional meristem (TM), flower meristem (FM), sympodial shoot meristem (SYM), vegetative meristem (VM), and inflorescence meristem (IM).

The flowering time, as well as the architecture of the inflorescence, determines the fruit yield. Due to the tomato’s sympodial shoot architecture, its shoot apical meristem (SAM) forms a transition meristem (TM), which results in a floral meristem (FM). At the same time, the flank retains the ability to form a new inflorescence meristem (IM). In addition, tomatoes can form new shoots from the axillary meristem (Figure 3) [23]. The genetic fundamentals of such complex processes are studied on characterized mutants with flowering disorder phenotypes. However, the mechanism of regulation and cross-interaction of genes underlying such phenotypes have yet to be well studied.

Under suitable conditions, plants transit from the vegetative phase to the reproductive phase. Plants produce florigens in response to endogenous and environmental stimuli. In tomatoes, the main florigen is considered to be SINGLE FLOWER TRUSS (SFT). The *sft* mutation produces a late-flowering phenotype and causes the replacement of flowers by vegetative shoots [24]. *SFT* is a member of the *CETS* gene family [25]. Their proteins bind to adapter proteins in the cytoplasm of SAM cells and translocate into the nucleus, forming florigen activation complexes through binding to the bZIP transcription factor [26]. SFT induces the expression of SAM-to-TM genes, particularly *FRUITFUL* (*FUL*) [27]. *SFT* is regulated by CONSTANS (CO) transcription factors and their homologs due to direct binding to its promoter region, and their suppression stimulates the development of flowers and fruits, which, as a consequence, leads to an increase in the tomato yield [28,29]. Evidence shows that signaling cystine-knot miniproteins (CMPs) interact with CO [30]. *CO* is regulated by the blue light receptor FKF1 (Flavin-binding Kelch repeat F-box 1) [31]. SQUAMOSA promoter-binding protein (SBP) is another factor that interacts with the *SFT* promoter region [32]. In addition, SBP-like proteins are directed by miR156 [33]. At the SAM, SBPs activate the expression of *FALSIFLORA* (*FA*) while DELLA and miR156-targeted SBPs activate *MACROCALYX* (*MC*) to promote inflorescence development [34]. In addition to floral meristem determinacy, miR156-SBP also regulates the locule number [35]. SBP13 inhibits the synthesis of cytokinins (CTKs), thereby suppressing the growth of lateral buds [36]. SBP15 regulates axillary bud development and growth by inhibiting auxin (AUX) transport and GOBLET (GOB) activity and interacts with BRANCHED 1b (BRC1b) to control abscisic acid (ABA) levels in axillary buds [37]. In addition, the miR171-GRAS module restrains the flowering time and trichome distribution by suppressing the activity of miR156-targeted SBP-like proteins [38]. FT-interacting proteins 1 (FTIP1) are involved in transporting the florigen signal [39]. SP acts mainly as an antiflorigen [40]. There is evidence that SP5G, SP5G2, SP5G3 [41,42], and SP3C [43] act as repressors of flowering, and SP3D/SFT as an activator [41]. It was also demonstrated that the night break and red-to-far-red light ratio are the reasons for the accumulation of *SFT*-like gene transcripts during the late-flowering stages of tomato [41]. Epigenetic modifications could regulate CETS because the ectopic expression of DNA demethylase in tomato had a similar phenotype to *CEN1.1*-overexpressing plants, manifested by the non-stable transition of meristems to IM, delayed growth, and increased number of leaves between inflorescences [44]. The balance between SFT and SP signaling is the main switch between the determinate and indeterminate development of meristem [45,46,47], and its possible regulatory mechanisms have been discussed previously [22].

*ANANTHA* (*AN*) and *FALSIFLORA* (*FA*) are considered inflorescence modifiers. The *an* mutant produces cauliflower-like inflorescences [48]. AN is an F-box protein involved in transcriptional co-activation with the transcription factor FA. *AN* activation occurs in the late stage of FM development [23]. The tomato *fa* mutation alters the development of the inflorescence, resulting in the replacement of flowers by secondary shoots, but also produces a late-flowering phenotype with an increased number of leaves below the first and successive inflorescences [49]. GAMYB could be involved in gibberellin-regulated flowering by activating FA gene transcription [50].

TERMINATING FLOWER (TMF), a transcription factor containing a conserved DNA-binding ALOG domain, maintains the meristem in a vegetative state. Single and multiplex knockout mutations of *TMF* and its paralogs, called *TMF FAMILY MEMBER*s (*TFAM*s), demonstrated the dominant role of TMF in the formation of condensates with other members of the *ALOG* family of genes [51]. These condensates bind to the *AN* promoter and suppress its expression at the vegetative meristem (VM) stage [52]. An exciting model for regulating the transition of SAM to FM has been proposed [53]. According to the source, naturally produced reactive oxygen species (ROS) contribute to the formation of transcriptional condensates of the TMF transcription factor proteins in tomato meristems due to the oxidation of their cysteine domains with the subsequent formation of disulfide bonds. TMF condensates sequester the *AN* locus, preventing its premature activation during meristem maturation, thereby regulating the transition to flowering. There is also evidence that TMF acts with cofactor BLADE-ON-PETIOLE (BOP) by forming a transcriptional complex [54] controlling the pleiotropic functions [55].

The absence of pedicel abscission phenotypes characterizes the natural mutants *jointless* (*j*) and *jointless*2 (*j2*). In addition, *j* has IMs that develop into vegetative growth, and *j2* has bifurcated inflorescences and sepals in the form of leaf-like structures. The *j2* phenotype is due to two independent mutations in the MADS-box gene *MBP21*, caused by the insertion of the *Rider* transposon and a single-nucleotide substitution that led to the appearance of a premature stop codon [56].

As suggested before, a MADS-box protein complex comprising at least J, MACROCALYX (MC), and MBP21 regulates pedicel abscission in tomatoes [57]. It was shown that MBP21 is involved in the ethylene and auxin regulatory pathway of sepal development [58]. It is also noted that one of the FANTASTIC FOUR family members, FAF1/2c reduces the stability of the COP9 signalosome, thereby regulating the expression of *SFT* and *J* [59].

At the TM and IM stages, three MADS-box genes of the *SEPALLATA 4* (*SEP4*) family are expressed—*J2*, *ENHANCER OF JOINTLESS 2* (*EJ2*, homolog of *MADS1*), and *LONG INFLORESCENCE* (*LIN*) [60]. Cross-interactions were detected between them, and their functional redundancy was confirmed in a collection of knockout mutants. Recently, the RNAi of tomato *SEP4*-like *CMB1* led to longer, branched, and indeterminate inflorescences that exhibited a transition from reproductive to vegetative growth and enlarged and abnormally fused sepals [61] and a yeast two-hybrid assay showed that CMB1 could interact with MC, J, and MBP21.

The *J2*/*EJ2* phenotype of excessive flowering and low fertility can be compensated by introducing a copy of the *SB* (*SUPPRESSOR OF BRANCHING*) locus, resulting in unbranched inflorescences [62]. It contains the *TM3* (*tomato MADS 3*) and *STM3* (*SISTER OF TM 3*) genes, which are antagonists of *J2* and *EJ2* [63]. Therefore, the regulation of flowering can be carried out by changing the dose of the antagonist factors, i.e., by increasing the copy number of their genes. There is cross-regulation between *STM3* and *J2* through direct binding, and their common target is FUL1 [64,65]. Recently, *TARGET OF EAT 1* (*TOE1*) from the *APETALA 2* (*AP2*) gene family was found to be a regulator of *STM3* [66].

*FUL*s are other MADS-box genes that play an important role in flowering. The *FUL* genes fall into two paralogous clades. Tomato has two genes in each clade: *FUL1* and *FUL2* in the former and *MBP10* and *MBP20* in the latter [67]. The analysis of CRISPR-Cas9 knockout plants showed that FUL2 and MBP20 promote the transition from vegetative to reproductive development and control inflorescence architecture [68]. At the same time, FUL1 most likely exhibits secondary functions during flowering, which the authors could not clarify. TM3/STM3 presumably controls floral transition by interacting with FUL2/MBP20 in a protein complex and repressing cytokinin inhibitors but subsequently exhibits antagonistic functions in determining FM and IM identity [69]. *MBP10* is expressed at weak to moderate levels, and its atypical short first intron lacks putative transcription factor binding sites, indicating possible pseudogenization [67].

The *MACROCALYX* (*MC*) gene belongs to the *APETALA 1*/*FRUITFULL* (*AP1*/*FUL*) subfamily of the MADS-box gene family and is closely linked to the *RIPENING INHIBITOR* (*RIN*) gene that regulates fruit ripening, see review [70]. The MC regulates sepal development, and the transcription factors J and EJ2 were recently shown to interact with MC directly [71,72]. It was demonstrated that MBP22 can form condensates with MC and the SEP proteins TM5 and TM29 [73]. *MC* is probably downregulated by *AGL* (*AGAMOUS*-like) genes [74,75]. TM5 is the only representative of the SEPALLATA3 clade, and it is involved in determining the identity of petals, stamens, and carpels in tomatoes [76]. TM5, together with other transcription factors like RIN, is involved in the regulation of fruit development [77]. TM5 may act altogether with TM29 [78]. It has also been shown that the expression of *AGL* and *TM*s is controlled by histone deacetylases [79] and WRKY factors [80].

Transcription factors containing a DNA-binding domain with one zinc finger, called DOF (DNA-binding with one zinc finger), regulate photoperiodic flowering. DOF10 is involved in the control of cell proliferation during the development of vascular tissue in flowers [81]. DOF9 is involved in inflorescence meristem control and floral meristem differentiation by regulating cell division genes and regulating the inflorescence architecture LIN [82]. Additionally, DDF1 has been shown to mediate circadian regulation via protein–protein interaction with the floral inducer SFT [83]. CDFs delay the flowering time by regulating various *FT*-like genes [84].

A reversal of flower development is an interesting event consisting of a change from floral to vegetative development. It has been shown that the silencing of the *GT11* gene, which belongs to the family of photosensitive transcription factors with a trihelix structure, leads to the formation of sepal-like petals in the second radial arrangement, carpel-like stamens in the third radial arrangement, and abnormal stem-like, leaf-like, and flower-like structures in the fourth radial arrangement in tomato [85]. These phenotypic manifestations, as well as the suppression of MADS-box gene expression, suggest the participation of GT11 in forming the pattern of floral organs and maintaining floral determinacy in tomatoes.

**Figure 4:**
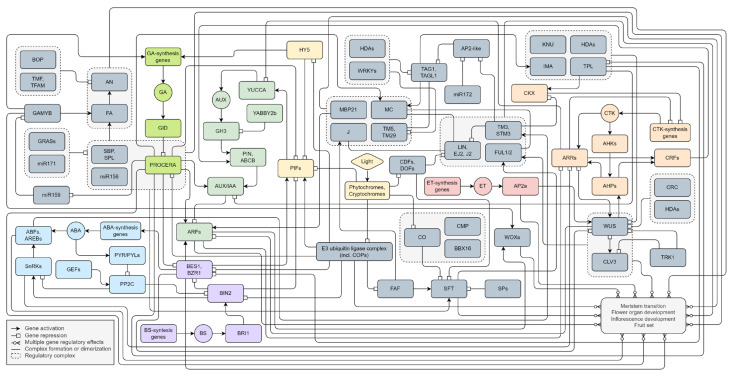
The flowering gene regulatory pathways in *S. lycopersicum*. All interactions are based on experimental data reported in scientific publications. A molecular interaction network model was created using the free online web application draw.io (www.drawio.com (accessed on 24 January 2024)).

We have presented a putative model of flowering factor regulatory pathways (Figure 4). The primary external stimulus to flowering is considered to be light signaling. It includes both the spectrum and the photoperiod. Plants perceive and respond to light signals via multiple sensory photoreceptors, including phytochromes and cryptochromes. The shared signaling molecules of these photoreceptors are the COPs E3 ubiquitin ligase complex and bHLH transcription factors PIFs. They trigger cascades of hormonal regulatory pathways, mainly AUX, GA, and CTK signaling, that activate meristem transition, inflorescence formation, and flower organ development. The most abundant transcription factors in such processes are MADS-box genes. It appears they form homo- or/and heterologous multimers and provide both the positive and negative regulation of tomato flowering. Due to overlapping targets, they may contribute to regulating flowering-related genes through dosage compensation. The disruption of such balance is possible due to other factors and cofactors by hormonal signaling. We assume three possible relationships of transcription factors: redundancy, additivity, and dependency. Redundancy is manifested in the functional identity of transcription factors. Additivity is associated with the provision of function through the joint contribution of each element. Direct dependence involves the activation or repression of the role of one factor only after interaction with another. Moreover, the autocatalytic regulation of the participants of regulatory cascades is possible.

## 3. Flower Development

**Figure 5:**
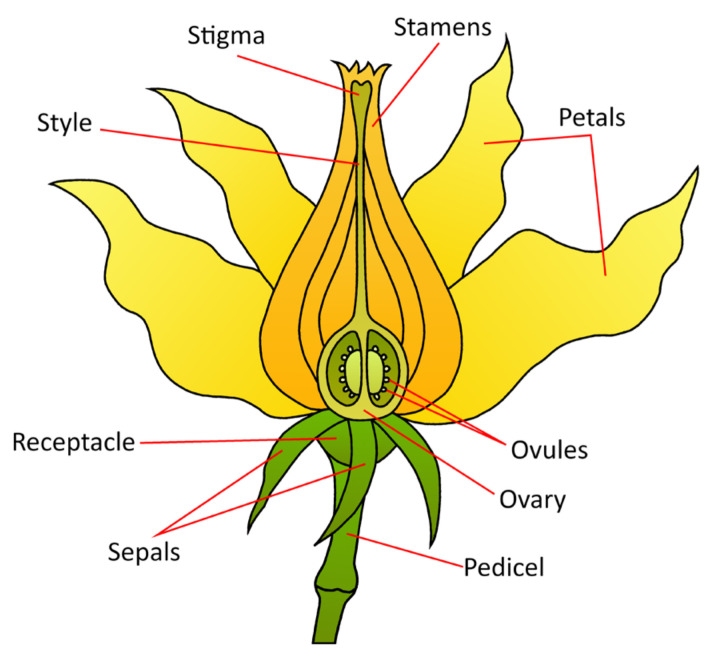
A stylized diagram of the tomato floral anatomy. This general morphology is consistent across angiosperms.

The bisexual tomato flower, like most angiosperm flowers, consists of four distinctive whorls of floral organs: sepals in the outmost whorl, petals in the second whorl, and stamens and carpels in the respective third and fourth whorls. The number and position of floral organs in each whorl are governed by floral homeotic genes and cadastral genes controlling the floral organ boundary (Figure 5).

The tomato fruit develops from the fertilized ovary, which is the broad base of the pistil. It is a berry consisting of a pericarp formed from the ovary wall, placenta, and pulp, containing seeds [86]. Therefore, many traits of fruit morphology are determined during flower development.

### 3.1. Flower and Fruit Abscission

**Figure 6:**
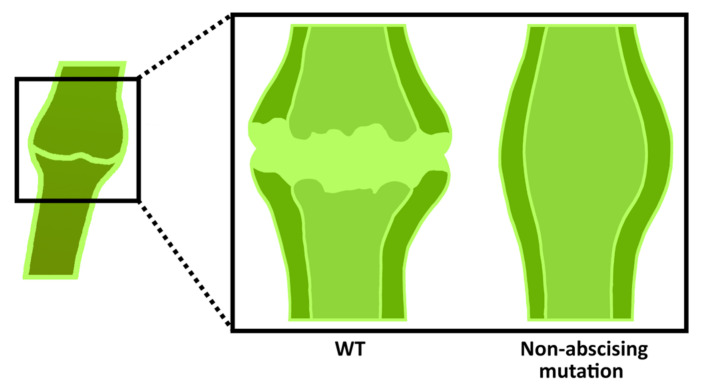
Representation of tomato abscission zone. It forms in the pedicels and has a knuckle-like structure in which a groove forms for abscission.

Plant reproductive organ rejection occurs in the abscission zone (Figure 6) and is one of the causes of low crop yields. It is triggered by developmental and environmental signals. An alteration in the auxin and ethylene contents in the abscission zone to a threshold value is known to result in abscission [87]. Abscission zone cells may remain in a non-dividing state without external stimuli and behave as meristematic cells [88]. Suppression of fruit abscission is also a valuable trait, leading to the convenience of tomato harvesting and processing. For example, in abscission mutants, pedicels and calyxes remain attached to the inflorescence axis, which reduces the mechanical damage to the fruit during transportation. Here, we present some recent studies of tomato genes conducting flower and fruit abscission.

*LSD* (*LESION SIMULATING DISEASE*) genes encode a family of zinc finger proteins essential in hypersensitive responses and programmed cell death induced by biotic and abiotic stresses. LSD and a bHLH-type transcription factor are involved in cytokinin-induced petal abscission via the regulation of *Aux*/*IAA* gene expression [89]. Evidence shows that the IDA (INFLORESCENCE DEFICIENT IN ABSCISSION) peptide and its IDA-like (IDL) homologs play a conserved and central role in this process. For example, the knockout of *IDL6* [90] suppressed *TAPG1* (polygalacturonase), *TAPG4*, and *CEL2* (cellulase), which is quite similar to the function of the phytosulfokine peptide. However, their regulation of abscission is different. The authors hypothesize that ethylene triggers IDA signaling, which promotes the expression of cell wall hydrolases that cleave polysaccharides in the cell wall and middle lamina, leading to abscission. Here, WRKY17 acts as a positive regulator of *IDL6* by directly binding to the W-box elements of its promoter. *IDA*-regulated genes are *KNOX*, which also control flower abscission. KNOX has been shown to inhibit the expression of the ethylene synthesis genes by directly binding to their promoters, and its ectopic expression in tomatoes suppresses flower abscission [91].

A lack of auxin induces the production of ethylene, which in turn is an abscission activator. Morphologically, this is characterized by the destruction of undifferentiated cells in the meristem to initiate their differentiation into the abscission zone. ILR and ILL hydrolyze amino acid-type IAA conjugates. Auxin is readily degraded in its free form, so the inactivation of the hydrolases of IAA conjugates may mediate delayed abscission [92].

Many transcription factors are involved in these processes. For example, it has been previously shown that the loss of MBP21 function denied abscission zone formation and the consequent separation of tomato fruit from the parent plant [56]. A *BELL* gene family member, *BL4*, is also involved in abscission zone formation [93]. It was confirmed by the anatomical analysis of the peduncle, after which it was evident that the zone was not formed in the *BL4*-RNAi lines, and more epidermal cell layers were observed at this location compared with WT. Also, the silencing of the intercellular auxin transporter *PIN1* accelerates flower abscission by increasing auxin accumulation in the ovule and decreasing the auxin content in the abscission zone [94]. In turn, its negative regulator is the transcription factor MBP9 [95].

ROS are involved in the biosynthesis and signaling of ethylene-dependent regulation. TIP (TONOPLAST INTRINSIC PROTEIN) aquaporins mediate the transport of ROS and water through the cell wall. Therefore, the suppression or overexpression of their corresponding genes shows delayed or accelerated abscission, respectively [96].

At the late stages of abscission, several key enzymes play an essential role in organ abscission. Cellulase and polygalacturonase are involved in cell wall degradation, and pectin methylesterase changes the chemical structure of the abscission zone through hydrolysis and causes cell wall and membrane degradation. However, the signaling proteins at this stage are hybrid products of the *PRP* (proline-rich protein) gene, which are involved in activating cell wall hydrolysis. In [97], as expected, the silencing of *HyPRP* (*HYBRID PRP*) resulted in delayed peduncle abscission.

P4H (prolyl 4-hydroxylase) catalyzes the posttranslational modification proline hydroxylation of cell wall hydrolases (TAPG and CEL) and expansins (EXPs). The RNAi of the *P4H3* gene showed decreased *TAPG*, *CEL*, and *EXP* transcript levels [98]. Phenotypically, this was manifested as a delayed abscission of tomato fruits in the latter stages of senescence.

Ultimately, we summarized the abscission-related studies on tomatoes from 2017 to 2023 in Table 1.

Despite the abundance of factors in the table, the main regulatory complex of abscission processes appears to be CLV-WUS (CLAVATA-WUSCHEL), which controls auxin and ethylene homeostasis in the abscission zone in response to external stimuli [108].

### 3.2. Ovary Development and Fruit Size

Fruit organogenesis begins in the early stages of flower development with the differentiation of carpels into locules, the number of which determines the final size of the fruit. Two loci, *fasciated* (*fas*) and *locule number* (*lc*), are responsible for their formation, and they affect the expression of *CLV3*, *WUS*, *YABBY2b*, and *TAG1* (tomato *AGAMOUS 1*) [112]. Previously, *YABBY2b* was reported as the candidate gene for the *fas* allele [113]. However, it was shown that CLV3 is associated with *fas* [112,114], while YABBY2b regulates auxin synthesis by suppressing *GH3.8*, which encodes indole-3-acetic acid-amido synthetase [115]. Meanwhile, WUS appears to mediate *lc*. The RNAi lines of the *WUS* gene confirmed the involvement of WUS in controlling the formation of floral organs and the number of locules in tomato fruits [116]. The expressions of *TAG1* and *CLV3* were changed in such lines.

*WUS* encodes a homeodomain transcription factor of the WOX (WUSCHEL-like homeobox) family. WOX controls the growth and development of plants by regulating the formation and maturation of stem cells in meristems. Null mutations in *WOX9* (related with natural mutation in *COMPOUND INFLORESCENCES*) and *WOX8* result in embryonic lethality, and mutations in the promoter region cause various defects in meristem development [117]. The knockout of the *WOX1* resulted in a phenotype with defects in the fusion of petals, carpels, and stamens, suggesting the involvement of the gene in the regulation of tomato flowering [118]. The knockout of the *WOX1* homolog *LAM1* (*LAMINA DELETION MUTANT 1*) also showed impairments in floral organ development, fruit size, secondary leaflet initiation, and leaf complexity [119,120].

There is evidence that *WUS* expression is regulated redundantly. For instance, *BRI1-EMS-SUPPRESSOR 1* (*BES1*) repressed the regulation ability of WUS via their heterodimerization, thus inhibiting WUS binding to the *CLV3* promoter [121]. *WUS* can also be repressed by KNUCKLES (KNU) through histone deacetylation [122]. The adapter proteins in this process are most likely INHIBITOR OF MERISTEM ACTIVITY (IMA). IMA was proposed to recruit KNU to form a transcriptional repressor complex with TOPLESS (TPL) and histone deacetylase [123]. In turn, IMA is induced by the MADS-box transcription factor AGAMOUS (AG) [124]. Moreover, CRABS CLAW (CRC) can interact with members of the chromatin remodeling complex that epigenetically represses *WUS* expression through histone deacetylation [125].

It was shown that the expression of *CLV3* and *WUS* appears to be restricted by the LITTLE ZIPPER protein (ZPR), encoded by the *DTM* (*DEFECTIVE TOMATO MERISTEM*) gene [126]. As suggested by the authors, *DTM* has negative feedback with HD-ZIP III homeodomain transcription factors. It has been established that the receptor-like cytoplasmic kinase TRK1 is necessary for maintaining the proper growth of meristems in tomato [127]. It turned out that *TRK1* silencing induced the formation of fasciated branches, altered inflorescences, and fruits of tomatoes with a significantly increased number of locules compared to wild-type plants. This phenotype, supported by further studies, is due to the interaction of TRK1 with CLV1, the receptor for the CLV3 peptide. In addition, TRK1 also interacts with and phosphorylates WUS. However, such cross-molecular interactions require further study.

Regulation of the formation of multiple locules in tomato fruits is achieved by modulating the levels of auxin and gibberellin (GA) in apical meristems, for which the transcriptional repressor TPL3 and WUS are responsible [128]. A protein interaction was found between them, ensuring the regulation of auxin transporter genes and enzymes for gibberellin biosynthesis. However, *CLV3* and *WUS* are not the only genes determining fruit size. It was previously shown that a representative of the AP2/ERF (ethylene responsive factor) superfamily of transcription factors, the ENO (EXCESSIVE NUMBER OF FLORAL ORGANS), regulates the activity of the floral meristem [129]. The authors demonstrated that *ENO* exhibits synergistic effects with mutations at the *fas* and *ls* loci. This is explained by the interaction of ENO with the *cis*-regulatory element of the *WUS* promoter. The clarified role of the AP2a factor was revealed in [130]. Loss-of-function mutants had a much higher ethylene production, leading the authors to infer a role as a negative regulator of ripening initiation, with deficient lycopene production and defects in chlorophyll degradation suggesting positive regulation during ripening. A CLV3-WUS module was shown to regulate auxin and ethylene homeostasis in low light-induced tomato flower abscission via the induction of the WUS target genes *KD1* (*KNOTTED1-LIKE HOMEOBOX PROTEIN*) and *FUL2* [108].

WUS is a bifunctional protein that can repress and activate gene transcription in SAM [131]. It induces CLV3 expression dose-dependently [132]. CLV3-mediated signaling through CLV1, CLV2-CRN, and BAM-RLK2 complexes restricts *WUS* expression [133,134]. In addition to the CLV-WUS complex, which determines the tomato fruit size, new regulatory elements are identified. For instance, a new tomato fruit size control module was recently disclosed [135]. Here, UV-damaged DNA-binding protein 1 (DDB1), which is a significant component of the Cullin4-RING E3 (CRL4) ubiquitin ligase complex, promotes the degradation of casein kinase (CK2). In turn, CK2 stabilizes cell division protein kinase (CDK2), acting as a positive regulator of fruit size.

**Figure 7:**
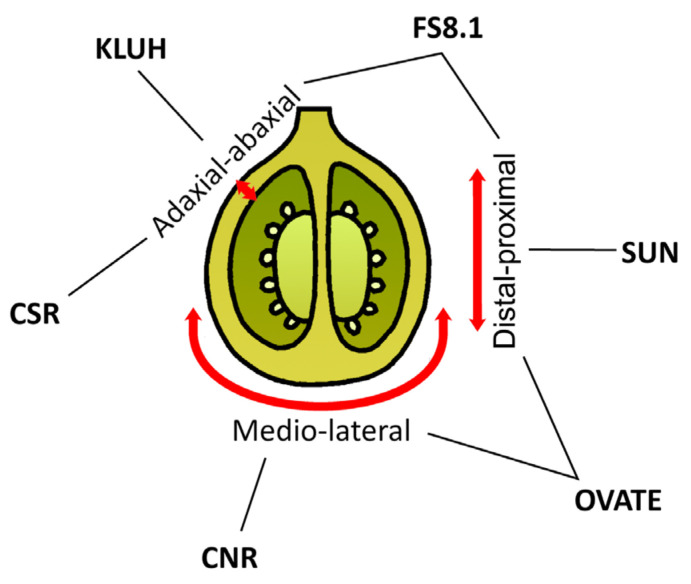
Genes influencing tomato fruit shape and weight. The red arrows indicate axes of growth: from proximal to distal, medio-lateral, and from adaxial to abaxial.

In addition to fruit size, the diversity of fruit shapes of tomato varieties is also attractive to consumers. Tomato loci responsible for fruit size are identified (Figure 7). *Fas* and *lc* control fruit locule numbers and their flat shapes, whereas *sun* and *ovate* control elongated shapes [136].

SUN and OVATE determine the shape of the ovary. Compared with WT, *ovate* primarily increases the fruit proximal end by increasing cell numbers in the proximal–distal direction and decreasing cell numbers in the medio-lateral direction, leading to a pear-shaped fruit [137]. OFPs (OVATE family proteins ) are a class of proteins with a conserved OVATE domain. OFPs interact with various transcription factors, including KNOX and BELL [93,138], and also directly regulate the expression of auxins, gibberellins [139,140], and ethylene-related genes [141]. The heterologous expression of *OFP1* and *MADS1* in the ovate mutant compensated for the elongated fruit phenotype [142]. The heterologous overexpression of citrus *OFP19* in tomatoes resulted in the pear-shaped ovary and fruit shape [143]. Transgenic tomato plants that overexpressed bottle gourd *OVATE1* had cone-shaped fruit, calyx hypertrophy, petal degeneration, and petal retention after flowering [144]. The overexpression of *OFP20* has been shown to regulate abscisic acid accumulation [145]. In addition, OFP20 may play an essential role in the crosstalk between brassinosteroids (BSs) and gibberellins [146]. On the other hand, the *sun* results in long fruit by increasing cell numbers along the entire proximal–distal direction in the pericarp and columella while decreasing cell numbers in the medio-lateral direction in the columella and septum [147].

TONNEAU1-recruiting motif proteins (TRMs) have been implicated in the control of organ shapes in tomatoes, which is contributed to via interaction with OFPs [148]. The *TRM1-5*-like genes promote fruit elongation, whereas *OFP1*-like genes play an antagonistic role [149]. The IQ67-domain (IQD) proteins regulate Ca^2+^ signaling and plant development through interactions with calmodulins, and SUN is one of them. SUN affects the expression of auxin-related genes [150,151] and brassinosteroids [140]. *SUN24* negatively regulates the expression of crucial abscisic acid signaling genes [152]. The ectopic overexpression of watermelon *IQD24* promoted tomato fruit elongation [153]. MAP70 (microtubule-associated protein) interacts with IQD21a to regulate tomato fruit shape [154]. It becomes clear that the interaction of OFP-TRM and MAP-IQD modules with microtubules lies in the physiology of fetal shape changes, as discussed in [155].

KNOTTED-like (KNOX, KN) proteins and BELL1-like (BLH) proteins belong to the same TALE homeodomain family. *KN2* from *CLASS*-*II KNOX* genes regulate fruit anatomy via gibberellin [156] and ethylene-dependent pathways [157]. The ectopic expression of litchi *KNOX* represses tomato flower abscission [91]. The heterologous overexpression of grape *KNOX63* in tomato induced smaller fruits and seeds than in wild-type or KN1-deficient plants [158]. There is also KN4, which affects pollen development via the regulation of GA and auxin genes [159]. BLHs have been recently shown to regulate chloroplast development and chlorophyll synthesis in tomato fruit [160,161]. KNOX and BLH proteins act together by forming heterodimer modules [162,163,164].

KLUH is a positive regulator of fruit size, and it was recently demonstrated that introducing point mutations into its promoter region can “improve” the attractive appearance of large fruits [165]. The duplication of *KLUH* and *STM3* and the corresponding increase in gene expression are decisive for *fw3.2*, one of the primary loci responsible for tomato fruit mass [63].

The *fw2.2 (fruit weight 2.2)* gene was found to control a significant QTL for tomato fruit size by negatively affecting cell numbers [166]. FW2.2 is a transmembrane protein containing a PLAC8 (PLACENTA-SPECIFIC 8) domain. Unfortunately, no functional analysis is yet available for FW2.2 [167]. However, like in the *sun*, the presence of a PLAC8 domain can be related to a putative function in regulating Ca^2+^ signaling [168].

In addition to *CNR* (*COLORLESS NON-RIPENING*)/*FW2.2* and *KLUH*/*FW3.2*, a new tomato cell size regulator gene was recently discovered—*CSR* (*CELL SIZE REGULATOR*)/*FW11.3* [169]. Its *CSR*-like 1/2/3 paralogs were also discovered. Co-expression analysis of these genes revealed a role for the *CSR* gene in cell differentiation during later stages of fruit development, including vascular development. The antagonistic roles of auxin and cytokinin could be related to CSR function, as several genes associated with these pathways were found in co-expression clusters. The authors also associate cell enlargement with increased endoreduplication.

Genes underlying the *fs8.1* locus that contribute to rectangular elongated tomato fruit have yet to be identified. Compared with *sun* and *ovate*, *fs8.1* showed a cellular patterning that was different from the effect of the other two genes: *fs8.1* led to increased fruit shape by increased cell number in the proximal–distal direction without a change in the medio-lateral direction [170]. Recently, the candidate genes of *fs8.1* have been proposed [171]. Its list includes genes encoding ERECTA (LRR receptor-like kinase), cytokinin oxidase, F-box protein SLOMO (SLOW MOTION), aminopeptidase protein, pentatricopeptide repeat protein, LRR domain-containing protein, and trihelix transcription factor. Thus, the *fs8.1* locule requires further investigation.

The *sun*, *ovate*, *fs8.1*, *kluh*, *csr*, and *cnr* loci display individual molecular mechanisms of ovary development in tomato. Although the ovary shape changes they exhibit differ, they can contribute synergistically by strengthening the effects of other mutations, allowing for the combination of these traits in breeding. In general, the effects of fruit shape loci are likely due to changes in the expression of genes related to phytohormones, cytoskeleton, and sugar transport and degradation genes.

## 4. Pollen Development and Fertilization

Developing functional pollen grains is a crucial aspect of plant sexual reproduction. The process involves three stages: microsporogenesis, postmeiotic microspore development, and microspore mitosis [172]. Pollen grains are developed from microspores in the microsporangium of an anther. The cell wall of a mature pollen grain is a multilayered structure consisting of sporopollenin-based exine [173] and cellulose-based intine [174,175]. Pollen development and maturation involve multiple cellular changes mediated via the precisely organized regulation of gene expression. They could be influenced by many factors, such as tapetum irregularity, cytoskeleton alteration, auxin metabolism aberration, altered sugar utilization, and ROS accumulation [176].

Fruit set depends on successful pollination and fertilization. Pollination involves the transfer of pollen grains from the anther to the pistil’s stigma. Fertilization requires the growth of the pollen tube in the pistil tissue to the ovary [177]. Fertilization of the ovary initiates the development of the ovary into a fruit [178].

### 4.1. Pollen Maturation and Sterility

During the growth and development of plant pollen, the autophagy of the tapetum provides the necessary nutrients for the development of microspores. Transcription factors induced by various signaling pathways act as molecular regulators in these processes. For instance, the MYB72 inhibits the autophagy in tomato anthers [179] while HB8, which is an HD-Zip III transcription factor, accelerates tapetum degradation [180]. Also, the knockout of genes encoding the bHLH transcription factors leads to dysfunctional meiosis and the development of an abnormal tapetum during flower development [181,182]. Moreover, SINA (SEVEN IN ABSENTIA) proteins with E3 ubiquitin ligase activity impact tomato floral structure [183].

Delayed or early tapetum degradation can affect pollen development, leading to male sterility. Genetic male sterility is attractive when obtaining hybrid seeds, ensuring high varietal purity. The genes that mediate male sterility are being actively studied. For example, the knockout of the *ABORTED MICROSPORES* (*AMS*) gene, encoding a bHLH transcription factor, led to critical changes in the morphology of tomato pollen grains and, as a result, their nonviability [184]. It has also been shown that the knockout of the strictosidine synthase gene STR1 results in abnormally small pollen grains with a structurally weakened exine, and the plants themselves do not set fruit after selfing [185]. In [186], it was demonstrated that the mitogen-activated protein kinase MPK20 positively regulates the development of mononuclear microspores during mid-to-late life. Silencing or null mutations of *MPK20* led to male sterility. In addition, the abortive nature of *MPK4*-silenced tomato pollen was confirmed in [187]. A dramatic increase in *ACO* expression occurs during pollen and seed maturation. The knockout of *ACO2* results in male sterility but reduces free proline levels [188].

ROS have been reported to act as regulators of anther development. RBOH (respiratory burst oxidase homolog) plays a key role in regulating ROS accumulation in anthers and mediates tapetum development [189]. Therefore, *RBOH*/*RBOHE* double knockout mutants exhibited complete male sterility, showing abnormal programmed cell death in the anthers. There is also evidence that the cytoplasmic invertase CIN7 is involved in pollen viability, which the authors also associate with increased ROS accumulation in *CIN7*-silenced tomatoes [190]. Meanwhile, the knockout of *IDA* (inflorescence deficient in abscission), which acts as the RLKs ligand, destabilizes ROS homeostasis that leads to a programmed cell death defect in the tapetum and septum and a failure of anther dehiscence [104]. The accumulation of ROS in anthers is related to heat shock transcription factors, which confers pollen thermotolerance [191].

Hormone-mediated regulation pathways of pollen development and maturation are actively studied. For instance, the study of the mediator complex is required to control the transcription of RNA polymerase II. The function of the MED18 (mediator complex subunit) subunit in pollen ontogeny has been reported [192]. The expression profiles of tapetum degradation genes and pollen maturation genes are in RNAi-silenced lines. There is also evidence that silencing *MED18* suppresses the expression of gibberellin biosynthesis genes, auxin transport genes, and regulators of leaf morphogenesis [193]. The importance of auxins in developing tomato anthers was demonstrated using the PIN8 transporter gene as an example [194]. Tomato lines with *PIN8* (*PIN-FORMED 8*) silencing had shortened anthers. They observed the abortion of microspores in anthers, low pollen fertility, and parthenocarpic fruits, which the authors attribute to a greater extent to the increased content of IAA conjugates in transgenes. There is also a clue that abscisic acid plays a role in the primary formation of pollen grains [195]. Ascorbic acid impairs tomato pollen fertility [196].

Meanwhile, salicylic acid has also been shown to impact pollen development [197]. At the same time, ethylene signaling is shown to modulate tomato pollen tube growth through modifications of cell wall remodeling and calcium gradient [198]. Moreover, tapetum degradation and pollen fertility are affected by brassinosteroid-mediated regulation [199]. Jasmonic acid (JA) facilitates flower opening and pollen maturation through the expression of MYB21 [200].

Because sepals and petals only support limited photoassimilates, pollen growth largely depends on the import of carbon resources. SWEET (SUGAR WILL EVENTUALLY BE EXPORTED TRANSPORTER) members are critical players in sugar allocation between source and sink organs [201], and it was shown that SWEET5b is required for pollen maturation in tomato [202]. Meanwhile, the overexpression of *Vitis vinifera* sucrose transporter *SUC27* in tomatoes resulted in longer petals and pistils, an abnormal stigma, and much less and shrunken pollen, while the *SUC11*- and *SUC12*-overexpressing lines had similar flower phenotypes compared with those of the wild type [203]. The silencing of hexokinase *HXK1* resulted in a decrease in flower numbers, increased rate of flower abscission, abnormal thickening of the anther wall, and reduced pollen and seed viability [111]. Here, it was shown that phytochrome-interacting factor 4 (PIF4) inhibited the transcriptional expression of *HXK1*.

The growth of a pollen tube requires the coordination of membrane receptor signaling, GTPase activity, and actin cytoskeleton assembly. Kinase partner protein (KPP) is a guanine nucleotide exchange factor, and it plays a crucial role in pollen tube growth by recruiting the actin-related protein complex to the membrane-localized receptors [204].

Tapetum cell formation requires a leucine-rich repeat (LRR) receptor kinase and its ligand TAPETUM DETERMINANT 1 (TPD1). Tomato *TPD1* loss-of-function mutants showed an alteration in redox homeostasis during male gametogenesis and are expected to regulate BES1, DYSFUNCTIONAL TAPETUM 1 (DYT1), DEFECTIVE IN TAPETAL DEVELOPMENT AND FUNCTION 1 (TDF1), and MYB33 [205]. In addition to LRR receptor kinases, there are lectin receptor kinases, which are also shown to have an essential role in the correct development and maturation of tomato pollen grains [206]. Here, it was shown that carboxypeptidase, cytochrome P450, and DNA mismatch repair proteins are associated with lectin receptor kinase activity.

PIFs act as central regulators in integrating light and temperature signals to optimize plant growth and development. It was shown that PIF4 regulates the anther’s adaptation to low temperature by directly activating *DYT1* expression [207]. Moreover, low temperature promotes the transcriptional activation of *TDF1* by the PIF4-DYT1 complex, thereby postponing tapetal autophagy [207]. There is also evidence that PIF4 could be a key transcription factor regulating *YUCCA* (*YUC*) genes, which are the main auxin synthesis genes, in tomato stamens [110]. For another PIF, it was shown that both glutamate synthase 1 (GLT1) and cell wall invertase 9 (CWIN9) involved in auxin and sugar homeostasis in anthers are directly regulated by PIF3 [208].

As for *MYB33*, its knockdown has been shown to restrict the expression of genes controlling flowering (*AN*, *FA*, *WOX9*, *SP*) and sugar metabolism genes (*CWIN*, sucrose-phosphate synthase, sucrose synthase, trehalose-6-phosphate synthase) [50]. The authors proposed that MYB33 contributes to brassinosteroid–gibberellin crosstalk in flowering. MYBs are commonly regulated by microRNAs. Therefore, they are also involved in the regulation of sporogenesis. The knockdown of *miR171* was reported to result in male sterility in tomatoes due to the production of small amounts of deformed and nonviable pollen due to delayed tapetum ontogeny and reduced callose deposition around tetrads [209]. Due to *GRAS24* being one of the miR171 target genes, it is a participant in gibberellin and auxin homeostasis regulation in pollen development [210]. MiR172, miR156, and miR160 are also shown to be involved in transcriptome remodeling during pollen development [211]. Epigenetic regulation is integral to transcript accumulation. DNA methyltransferase *CMT4*, which is actively expressed during the flowering and early fruit development stages of tomato, has been shown to activate the genes of pollen wall development and pollen tube elongation [212].

The diversity of regulatory genes responsible for anther cell differentiation and pollen formation illustrated here allows us to characterize the gene crosstalk occurring in tomato and other crops. Although a complex system of gene expression and interactions, the molecular network of anther and pollen development is highly conserved. It involves various hormones and transcriptional and epigenetic factors. They regulate gene expression involved in tapetum degradation, cytoskeleton rearrangement, sugar metabolism, transport, and ROS accumulation.

### 4.2. Self- and Cross-Incompatibility

Modern domesticated tomatoes have been derived from many consecutive selections, resulting in a partial loss of genetic diversity. One way to expand the pool of available genes in tomato to achieve new desirable agronomic traits or for fundamental purposes is to obtain hybrids with its wild relatives. In this case, the problem of interspecific incompatibility arises. The physiological basis of interspecific incompatibility is still emerging. The nature of tomatoes’ reproductive barriers includes prezygotic and postzygotic isolating mechanisms. Tomato wild relatives are characterized by various reproductive biology systems [213], including self-compatible and self-incompatible taxons and facultative and unilateral incompatibility.

Most wild tomato plants cannot self-pollinate due to the protruding stigmas on the flowers. The elongated style prevents pollen grains from becoming trapped on the stigma, resulting in male sterility. In this case, high temperature is a prerequisite for lengthening the column, activating a shift in the hormonal balance. *LST* (*LONG STYLES*) is identified as a candidate gene mediating the elongation phenotype [214]. It encodes the ethylene receptor protein and is homologous to the *Arabidopsis EIN4* (*ETHYLENE-INSENSITIVE 4*) and tomato *ETR5* (*ETHYLENE RECEPTOR 5*) genes. Its overexpression reduced the severity of the style elongation phenotype in male-sterile tomatoes. The genes controlling the conversion from flush stigmas to inserted stigmas were identified in [215]. They appear to be C2H2-type zinc finger transcription factors—one controls the conversion of exserted to flush stigmas, while the other regulates the conversion of flush to inserted stigmas.

The callose wall surrounds the sporocytes while meiosis occurs. The temporary isolation of the sporocyte may be connected with the sporocyte’s differentiation process. Callose accumulates in the walls of incompatible pollen grains and tubes [216,217]. It has been shown that the β-1,3-glucanase encoding gene (*BG10*) regulates pollen development and seed production by modulating callose deposition [218].

MSH2 (MutatorS-Homolog 2) is involved in recognizing and repairing DNA errors. The suppression of *MSH2* gene expression in [219] using RNAi resulted in significant phenotypic abnormalities. This suppression contributed to the disruption of the light-dependent repair of thymine dimers. *MSH2* silencing also affected the progression of male meiosis, either arresting at the zygotene stage or forming diploid tetrads. Thus, MSH2 may be a ploidy regulator in addition to its reparative function. Indeed, using *MSH2* silencing, it was possible to establish that changing the lighting period may regulate the frequency of meiotic recombination within certain limits [220].

An example of overcoming interspecific Solanum incompatibility is given in [221]. The overexpression of the farnesyl pyrophosphate synthase gene FPS2 from *Solanum pennellii* in *S. lycopersicum* has been shown to compensate for the pollen incompatibility phenotype of the latter. The authors propose that FPS activity is required to prenylation a specific pollen rejection factor that interacts with the corresponding pistil barrier factor in *S. pennellii*. The *S. pennellii* barrier factor gene was also identified [222]. It has been shown that CRISPR/Cas9 mutations in the *DIR1*-like (*DEFECTIVE IN INDUCED RESISTANCE 1*) gene of *S. pennellii* allow *S. lycopersicum* pollen tubes to grow to the lower third of the style.

In summary, there are several reasons for incompatibility. Flower size and the distance the stigma extends beyond the anther determine the chance of self-fertilization. The size of the pollen grains is also important, as larger grains contain more nutrients, allowing the pollen tubes to grow to longer lengths. The molecular mechanisms of pollen rejection are also affected. At the postzygotic stage, incompatibility is caused by the disruption of the conjugation and segregation of homologous chromosomes due to their structural differences and epigenetic modifications. The genetic basis of these processes *is poorly known* and needs further study.

### 4.3. Parthenocarpy

Seed formation is the most crucial stage in fruit development; despite this, seedless fruits and fruits with underdeveloped or few seeds are found in both wild and cultivated tomatoes. This is possible due to parthenocarpy, in which the fruits develop without fertilization, and asthenospermia, in which pollination and fertilization are necessary. However, the embryos cannot form or are aborted before the seeds are formed. The complete penetration of pollen tubes can initiate fruit set independently of fertilization by activating genes regulating cell division and expansion [223]. Exposure to extreme conditions results in the suppression of fruit set due to low pollen viability. Seedless fruits are not only interesting from a developmental point of view. However, they are also a desirable agronomic trait due to their high soluble solids content and the lack of need to separate the seeds for cooking. Parthenocarpy has several advantages for growers, including avoiding emasculation in F1 hybrids and reducing the threat of heat stress during fruit set [224]. Parthenocarpy can increase winter and early yields, allowing for tomato harvests all year round [225]. In addition, the postharvest storage time of seedless fruits is longer than that of seeded fruits because seeds produce senescence hormones [226]. Parthenocarpy can be induced forcibly by external stimulation with plant hormones [227,228]. However, this may lead to undesirable pleiotropic effects, so studying the molecular basis of parthenocarpy is a priority.

The regulation of parthenocarpy is presumably carried out by auxin and gibberellin signaling cascades. The auxin and gibberellin regulatory pathways interact hierarchically and are the primary hormones that promote fruit set [229]. Auxin and gibberellin signaling cascades are negatively regulated by the DELLA component (*GRAS* gene encodes protein-containing “D-E-L-L-A” amino acid sequences) and the ARF7/IAA9 complex (auxin response factor/IAA), and cross-signaling between them controls the initiation of maturation fruits. As was shown, the inhibition of any of the components in these interactions leads to the appearance of seedless tomato fruits [230]. IAA9 is involved in the control of ARFs, and its suppression in tomato induces parthenocarpy [231].

Based on the assumption that genes of the *TOPLESS* (*TPL*) family are involved in auxin-mediated signals in the ovary, the authors silenced *TPL1* in tomatoes [232]. These plants did not exhibit pleiotropic effects under normal conditions and produced seedless fruits upon flower emasculation and heat shock, which was associated with changes in cytokinin levels. TPL1 interacts with IAA9, and a mutation of the *IAA9* gene leads to parthenocarpic fruit formation [233,234].

DELLA proteins localized in the nucleus are negative growth regulators. DELLA is encoded by the *PROCERA* gene, and its loss of function in the homozygous state results in dwarfism [235] and parthenocarpy [236]. DELLA proteolysis is mediated by the gibberellin-activated GID receptor [237]. The binding of gibberellin to the GID receptor increases the affinity of the latter for DELLA [238].

MADS-box factors are another participant in signaling regulation mediating parthenocarpy [1]. Indeed, seven MADS-box genes were previously reported to be expressed during flower development and the early stages of fruit and seed development [239]. Here, it was hypothesized that ovary and fruit development are a continuation of the flower development program. The MADS-box factors TAG1 and TAGL1 (tomato AGAMOUS-like 1) are involved in tomato fruit set [240,241] and have redundant and divergent functions [75,242]. Some other members of the MADS-box factor family are also involved in regulating tomato reproduction. For example, in [243], the authors found that the mutated MADS-box alleles of the *AGL6* gene ensure the tomato yield under heat stress conditions. CRISPR/Cas9-mediated mutations in *AGL6* produce facultative parthenocarpy, manifested by the development of seedless fruits comparable in weight and shape to wild-type fruits. One of the genes with increased expression induced by fertilization is the cell proliferation regulator cytochrome P450 *KLUH* gene. The ectopic overexpression of *KLUH* in tomato stimulated both integument growth in unfertilized ovules and parthenocarpy, indicating that its suppression by AGL6 is of primary importance for preventing fertilization-independent fruit set [244]. Also, silencing *AGL6* resulted in abnormally fused sepals and smaller, light green petals [74]. In such lines, *MC*, which is involved in the development of sepals, and *GOB* (*GOBLET*), which affects the initiation of the formation and division of leaf blades, were suppressed. *AGL11* gene expression correlates with early fruit development. Thus, the phenotypes associated with *AGL11* resemble those of other representatives of the MADS-box—*TAG1* and *TAGL1* [245]. In addition, metabolic reprogramming was observed to occur in sepals and fruits, with strong effects on cell wall-related genes.

Parthenocarpy is often accompanied by sterility due to either defects in the pollen or changes in the ovary. For instance, the formation of parthenocarpic fruits in the *hydra* mutant is associated with the lack of development of both male and female sporocytes. It was established that the *HYDRA* gene encodes the tomato ortholog of SPOROCYTELESS (SPL). The connection with sporogenesis in tomatoes was confirmed in RNAi lines and *hydra* lines overexpressing *SPL*/*HYD* [246]. In this case, parthenocarpy is explained by an increase in the level of expression of auxin and gibberellin genes in the ovary. SES (SEXUAL STERILITY) is another SPL homolog exhibiting male and female sterility [247].

Other actors in regulatory pathways are miRNAs. The authors of [248] showed that miRNAs’ modulation of hormone-dependent transcription factors affects the development of ovules and fruit set. Indeed, they found changes in the expression dynamics of the *miR159*/*GAMYB* system during the early stages of fruit development. Thus, the overexpression of the gene encoding the miR159 precursor in tomato suppressed *GAMYB* genes in developing ovaries, which led to earlier fruit initiation and parthenocarpy. Altered responses to auxins and gibberellins explained this. The above conclusions were confirmed later [249]. The overexpression of *GAMYB2*, a significant target of miR159, resulted in a flat fruit phenotype, while the loss of *GAMYB2* function had the opposite effect, resulting in smaller and elongated fruits. This regulation was mediated mainly via the direct repression of the *GA3ox2* gene. Confirmation of the connection between the parthenocarpic phenotype and disturbances in the expression of auxins and cytokinins in flowers is given in another publication [250]. Here, parthenocarpy was caused by a loss-of-function mutation in the gene encoding a receptor-like protein kinase expressed in vascular bundles of young buds. The mutation resulted in the increased expression of the gibberellin metabolism gene *GA20ox1*. Thus, MYB21 is a negative regulator of parthenocarpic fruit development, exerting regulation directly or through the JA signaling pathway [251].

The spectrum of genes responsible for parthenocarpy is broad. Thus, we summarize related studies on it from 2017 to 2023 in Table 2.

Thus, auxins, gibberellins, and homologous MADS-box transcription factors are the most critical players in regulating fruit set. Unfortunately, a clear picture of the regulation has not yet been formed due to the redundancy of MADS-box factors showing both positive and negative regulation in tomato fruit development, as well as the possible existence of other actors involved in this process, such as brassinosteroids, abscisic acid, jasmonates, and salicylates (SAs). For example, cytokinins induce parthenocarpy by modulating GA and AUX metabolism [266]. In turn, ABA acts as an antagonist of GA and AUX by inhibiting ovary development [267,268]. Ethylene suppresses tomato fruit set by stabilizing DELLA repressors [269]. BSs can increase ethylene production in tomato fruit, so they should stimulate parthenocarpy. However, their exogenous application on tomato flowers does not produce a seedless phenotype [270]. Regarding JA, there is evidence that a loss-of-function mutation of the lipoxygenase gene leads to the formation of parthenocarpic fruits in *Cucurbita pepo* [271]. Presumably, the increased expression of SA genes should favor the formation of seedless fruits due to their antagonistic nature toward JA. These findings provide fertile ground for further studies of the regulatory systems controlling fruit development.

## 5. Conclusions and Future Prospects

The number of studies on regulating plant life-cycle processes conducted on model objects such as tomatoes continue to increase. Recently, significant progress has been made in understanding the systems and processes of signal transmission. The discovery of gene functions and the regulation systems of tomato reproduction systems allows us to better understand the biology of the processes under consideration of their fundamentals. This knowledge also has a high potential for use in the applied aspect of developing agricultural plant varieties with improved properties.

In the future, the search for new candidate genes involved in tomato reproductive signaling cascades will predominantly be carried out using genetic and bioinformatic methods, possibly leading to the discovery of other groups of regulatory signaling pathways. There is still no clear understanding of their signaling regulation systems. Only now is the functional redundancy of transcription factors involved in flowering becoming apparent. Understanding the “dark horses” of regulatory cascades, such as ABA [195,268] and BSs [199,272,273], is also expanding. The ideas about the influence of epigenetic modifications [125,274,275], ncRNAs [248,262,276,277,278], and external factors [3,279,280] on signaling regulation have been greatly expanded recently. Modern genetic engineering approaches, including targeted genome editing using CRISPR/Cas9 technology, the use of base [281,282] and prime editing [283,284] for precision gene correction, application of omics technologies, and the collection and processing of bioinformatic data by new powerful algorithms are helping researchers to solve problems in this area.

Some limitations should be taken into account when interpreting new data. For example, orthologous genes are unreliable predictors of expression in different species because they often have different regulatory mechanisms. Simplified laboratory experimental conditions cannot establish all possible gene regulation and expression subtleties because reproductive processes are discrete and occur in different tissues at certain life-cycle stages. The studied gene regulators are often represented by large families of gene paralogs, which impose objective difficulties in understanding the function of each of them; confusion in gene numbering is a common phenomenon for such studies.

As a complementary approach in agriculture, growing crops under urban conditions is an option. So-called “urban farms” require crop varieties that are both compact and fast-growing. Although the yield of such plants may be lower, this can be compensated for by growing plants at higher densities, thus maintaining productivity in limited space. This direction has also been taken for tomatoes, e.g., in [42]. CRISPR/Cas9 mutations in *SP5G* and *SP* induced rapid flowering and enhanced the compact determinant growth in tomato. Consequently, this resulted in early harvest. Similar results were obtained in [285], where, in addition to creating double *SP5G*-*SP* knockouts, triple deficient mutants *SP5G*-*SP-ER* (where *ER* is the gene controlling internode length) were obtained, showing even greater compactness. Increased fruit size combined with determinant growth is a promising approach for tomato cultivation under urban conditions [286,287]. Thus, in addition to *SP5G*-*SP* null mutants, mutations can be made in the regulatory regions of *CLV3* and *WUS* genes, thereby increasing the number of locules in fruits. In [288], the tomato was edited for six loci: general plant morphology (*sp*), fruit shape (*ovate*) and size (*fas* and *fw2.2*), number of fruits (*multiflora*), and nutrient composition (lycopene beta cyclase gene). The authors obtained 15 combinations of independent alleles with a loss of function of the target genes. In the future, the discovery of new genes associated with improved agronomic traits is potentially compatible with existing genes.

Producers tend to avoid the exogenous application of hormones to stimulate specific physiological processes in plants in favor of controlling the endogenous regulatory systems, which is explained by the economic benefits, lower labor costs, and the possibility of promoting their products as eco-friendly. In addition, physiological changes in such plants have little or no undesirable pleiotropic effects. As for the practical application of the study of the considered reproduction genes, these are “golden opportunities” in overcoming interspecific incompatibility [62,221,222], pollen fertility [186,196], changing the size of the fruit due to changes in the number of locules and pericarp growth [129,165,289], changing the number of fruits due to an increasing number of inflorescences [119,290], and much more. All this is possible by inserting expression cassettes into the genome, i.e., developing genetically modified (GM) plants. Currently, society has not formed a clear opinion on the safety of GM plants. Thus, there are several risks associated with them. Cross-pollination ensures the flow of genes between populations and related species. In particular, the spread of transgenes through pollen can lead to the introgression of marker genes, whether antibiotic or herbicide-resistance genes, into the weed genome, leading to the emergence of herbicide-resistant weeds [291]. A possible solution to these problems lies in studying flowering genes, particularly those associated with the sterility trait of genetically modified crops. In addition, available modern genetic engineering methods for crop improvement allow for the selection of plants that do not contain foreign genes [292]. Obtaining plants with edited genomes makes it possible to bypass legal restrictions on the production and distribution of GM organisms in some countries [293].

This review has considered recent advances in studying tomato reproductive factors using different genetic approaches. Current research on tomato reproductive factors continues to expand the understanding of the molecular basis and physiological mechanisms of these processes, which opens up new opportunities for the practical application of this knowledge. We hope the new data presented here will add to the existing mechanisms describing the regulation of tomato reproductive systems.

## Figures and Tables

**Table 1:** Recent studies of tomato genes that facilitate flower abscission.

Gene	Function	Method	Reference	Year
*PIN1*	Encodes an auxin efflux carrier; involved in the maintenance of embryonic auxin gradients	RNAi silencing	[94]	2017
*J2*, *EJ2*	Encode a member of the R2R3-MYB transcription factor gene family; regulate floral organ and seed development	CRISPR/Cas9 knockout	[60]	2017
*MBP21*	Encodes a member of the R2R3-MYB transcription factor gene family; regulates floral organ and seed development	CRISPR/Cas9 knockout	[56]	2017
*HyPRP*	Encodes a putative cell wall protein consisting of a repetitive proline-rich N-terminal domain and a conserved eight-cysteine motif C-terminal domain that is involved in signaling events	RNAi silencing	[97]	2018
*J*	Encodes a MADS-box factor controlling abscission zone development	Heterologous expression (*Pyrus sinkiangensis*)	[99]	2018
*EBF3*	Encodes factor of ubiquitin–protein ligase complex that represses ethylene action and promotes growth by directing EIN3 degradation	RNAi silencing, OE	[100]	2018
*PP2C1*	Counteract mitogen-activated protein kinase pathways; co-receptor for the phytohormone abscisic acid	RNAi silencing	[101]	2018
*P4H3*	Encodes a prolyl-4 hydroxylase that can hydroxylate proline-rich peptides; involved in cell division and expansion	RNAi silencing	[98]	2019
*ILL*	Involved in auxin metabolic process	VIGS	[92]	2019
*PYL9*	Encodes protein that functions as abscisic acid sensor	RNAi silencing, OE	[102]	2019
*J2, EJ2*	Encode a member of the R2R3-MYB transcription factor gene family; regulate floral organ and seed development	CRISPR/Cas9 knockout	[62]	2019
*NCED1*	Encodes a 9-*cis*-epoxycarotenoid dioxygenase; involved in carotene catabolic process	OE	[103]	2019
*KNAT1*	Involved in meristem formation	Heterologous expression (litchi)	[91]	2020
*IDA*	Encodes a small protein that is involved in floral organ abscission, regulation of cell diameter	CRISPR/Cas9 knockout	[104]	2020
*IDL6*	Encodes a small protein that is involved in floral organ abscission, regulation of cell diameter	CRISPR/Cas9 knockout	[90]	2021
*TIP1;1*	Act as functional channels for water, urea, glycerol, H_2_O_2_, and CO_2_ transport across cell membranes	CRISPR/Cas9 knockout	[96]	2021
*BL4*	Involved in the regulation of meristematic activity via regulation of DNA-templated transcription	RNAi silencing	[93]	2021
*KD1*	Involved in meristem formation	RNAi silencing	[105]	2021
*GNT1*	Encodes an enzyme-initiating complex N-glycan maturation in the Golgi apparatus	RNAi silencing	[106]	2021
*ILR1*	Encodes an IAA–amino hydrolase	Heterologous expression (*Prunus persica*)	[107]	2021
*BAM1*	Encodes a CLV1-related receptor kinase-like protein required for both shoot and flower meristem function	CRISPR/Cas9 knockout	[108]	2022
*CLEs*	Encode a group of small secretory peptides, which regulate cell proliferation and differentiation	VIGS, CRISPR/Cas9 knockout		
*CLV1/3*	Control shoot and floral meristem size, and contribute to establishing and maintaining floral meristem identity	RNAi silencing, CRISPR/Cas9 knockout		
*WUS*	Homeobox gene controlling the stem cell pool	RNAi silencing, OE		
*FUL1/2*	MADS-box genes that are involved in fruit development, maintenance of inflorescence meristem identity, regulation of flower development	RNAi silencing, OE		
*HB15A*	Encodes HD-ZIP III protein; transcriptional regulator	CRISPR/Cas9 knockout	[109]	2022
*FZYs*	Encode a flavin monooxygenases that are involved in the auxin biosynthesis pathway	VIGS	[110]	2023
*LOL1*	Involved in cell death processes	RNAi silencing	[89]	2023
*HXK1*	Acts as sugar sensor that may regulate sugar-dependent gene repression or activation	RNAi silencing	[111]	2023

**Table 2:** Recent studies of tomato genes that facilitate parthenocarpy trait.

Gene	Function	Method	Reference	Year
*AGL6*	Encodes MADS-box transcription factor; gene regulator	CRISPR/Cas9 knockout	[243]	2017
*IAA9*	Regulator in auxin signaling	CRISPR/Cas9 knockout	[234]	2017
*miR159*	Encodes small ncRNA that modulate gene expression	OE	[248]	2017
*SPL/HYD*	Encodes MADS-box transcription factor; gene regulator	VIGS	[246]	2017
*TIR1/AFB2*	Encode auxin receptors that mediate auxin-regulated transcription	Heterologous expression (*Cucumis sativus* L.)	[252]	2017
*DELLA*	Regulator in gibberellic acid signaling	Complementation	[236]	2018
*POD1*, *MED18*	A coactivator for DNA-binding factors that activate transcription via RNA polymerase II	CRISPR/Cas9 knockout	[192]	2018
*ARF5*	Mediates auxin responses via expression of auxin regulated genes	Silencing by amiRNA	[253]	2018
*IAA9*	Regulator in auxin signaling	RNAi silencing	[233]	2019
*RLK*	Encodes a transmembrane receptor protein	RNAi silencing	[250]	2019
*MET1*	Encodes a cytosine methyltransferase; epigenetic regulation	CRISPR/Cas9 knockout	[254]	2019
*SPMS*	Catalyzes the production of the linear triamine, spermidine, from putrescine	Heterologous expression (yeast)	[255]	2019
*DOF10*	Gene regulator; involved in plant development and defense regulatory networks	RNAi silencing; OE	[81]	2019
*AP3*	Transcription factor involved in the genetic control of flower development	RNAi silencing	[256]	2019
*NCED1*	Encodes a 9-*cis*-epoxycarotenoid dioxygenase; involved in carotene catabolic process	OE	[103]	2019
*GA20ox2*	Involved in gibberellin biosynthesis	Heterologous expression (*Pyrus bretschneideri*)	[257]	2020
*PAD1*	Encodes an aminotransferase; involved in auxin homeostasis	RNAi silencing	[258]	2020
*HB15A*	Encodes HD-ZIP III protein; transcriptional regulator	CRISPR/Cas9 knockout	[259]	2020
*FRK2*	Involved in fructose metabolic process and starch biosynthetic process	RNAi silencing		
*TPL1*	Transcriptional co-repressor	RNAi silencing	[232]	2021
*KLUH*	Cytochrome P450 CYP78A monooxygenase subfamily member; cell proliferation	OE	[244]	2021
*IAA9*	Regulator in auxin signaling	CRISPR/Cas9 knockout	[260]	2021
*HWS*	Encodes F-box protein that is involved in regulation of gene silencing by miRNA	Heterologous complementation (*Arabidopsis thaliana*)	[261]	2021
*HB15A*	Encodes HD-ZIP III protein; transcriptional regulator	CRISPR/Cas9 knockout	[262]	2021
*CYP78A6*	Cytochrome P450 CYP78A subfamily member; seed development	Heterologous expression (*Pyrus bretschneideri*)	[263]	2021
*CIN7*	Catalyzes the hydrolysis of sucrose into glucose and fructose for the supply of carbohydrates to sink organs via an apoplastic pathway	RNAi silencing; OE	[190]	2021
*DELLA*	Regulator in gibberellic acid signaling	Target-AID	[264]	2022
*ARFs*	Mediate auxin responses via expression of auxin-regulated genes	CRISPR/Cas9 knockout	[265]	2023
*IAA9*	Regulator in auxin signaling			
*POD2*	Encodes the G-type lectin receptor kinase; plant development and responses to stresses	RNAi silencing	[206]	2023

## Data Availability

Not applicable.

## Abbreviations

ABA, abscisic acid; ABC, ATP-binding cassette transporter; ACO, acotinase; AFB2, auxin signaling F-box 2; AG/AGL, AGAMOUS/AGAMOUS-like; AHK, Arabidopsis histidine kinase receptor; AHP, Arabidopsis histidine phosphotransfer protein; ALOG, Arabidopsis LSH1 and Oryza G1; AMS, ABORTED MICROSPORES; AN, ANANTHA; AP, APETALA; ARF, auxin response factor; ARR, Arabidopsis response regulator; AUX, auxin; BAM1, BARELY ANY MERISTEM1; BBX, B-box domain protein; BES1, BRI1-EMS-SUPPRESSOR 1; BG, β-1,3-glucanase; bHLH, basic helix–loop–helix domain; BIN, BRASSINOSTEROID-INSENSITIVE; BL/BLH, BELL/BELL-homolog; BOP, BLADE-ON-PETIOLE; BRC, BRANCHED; BRI1, BRASSINOSTEROID INSENSITIVE 1; BS, brassinosteroid; bZIP, basic leucine zipper domain; BZR1, BRASSINAZOLE-RESISTANT 1; C2H2 zinc fingers, Cys2-His2 domain; CDF, cycling DOF; CDK, cell division protein kinase; CEL, cellulase; CEN, CENTRORADIALIS; CETS, CEN/TFL1/SP; CIN, cytoplasmic invertase; CK, casein kinase; CLE, CLV3/EMBRYO SURROUNDING REGION; CLV, CLAVATA; CMB1, carnation MADS-box 1; CMP, cystine-knot miniprotein; CMT, chromomethylase; CNR, COLORLESS NON-RIPENING; CO, CONSTANS; COP, CONSTITUTIVE PHOTOMORPHOGENIC; CRC, CRABS CLAW; CRF, cytokinin response factor; CRISPR, clustered regularly interspaced short palindromic repeats; CRN, CORYNE; CSR, CELL SIZE REGULATOR; CTK, cytokinin; CWIN, cell wall invertase; CYP, cytochrome P; DDB, DNA damage-binding protein; DDF1, DOF DAILY FLUCTUATIONS 1; DIR1, DEFECTIVE IN INDUCED RESISTANCE 1; DOF, DNA-binding with one zinc finger; DTM, DEFECTIVE TOMATO MERISTEM; DYT1, DYSFUNCTIONAL TAPETUM 1; EBF3, EIN3 binding F-box; EIN, ETHYLENE INSENSITIVE; EJ2, ENHANCER OF JOINTLESS 2; ENO, EXCESSIVE NUMBER OF FLORAL ORGANS; ER, ERECTA; ERF, ethylene responsive factor; ET, ethylene; ETR, ethylene receptor; EXP, expansin; FA, FALSIFLORA; FAF, FANTASTIC FOUR; FAS, FASCIATED; FKF1, Flavin-binding Kelch repeat F-box 1; FM, floral meristem; FPS, farnesyl pyrophosphate synthase; FRK, fructokinase; FS, FRUIT SHAPE; FTIP, FT-interacting protein; FUL, FRUITFUL; FW, FRUIT WEIGHT; FZY, FLOOZY; GA, gibberellin; GA_ox, gibberellin oxidase; GAMYB, gibberellin-associated MYB; GEF, guanine exchange factor; GH3, GRETCHEN HAGEN 3; GID1, GA INSENSITIVE DWARF 1; GLT, glutamate synthase; GM, genetically modified; GNT, N-acetyl-glucosaminyltransferase; GOB, GOBLET; GRAS, GIBBERELLIC-ACID INSENSITIVE/REPRESSOR OF GAI/SCARECROW; GT, galactosyltransferase; GTPase, guanosine triphosphate hydrolase; HB, homeobox; HD-ZIP III, class III homeodomain-leucine zipper protein; HWS, HAWAIIAN SKIRT; HXK, hexokinase; HY, ELONGATED HYPOCOTYL; HYD, HYDRA; HyPRP, hybrid PRP; IAA, indole-3-acetic acid; IAA9, IAA INDUCIBLE 9; IDA, INFLORESCENCE DEFICIENT IN ABSCISSION/INFLORESCENCE DEFICIENT IN ABSCISSION-LIKE; ILR/ILL, IAA-LEUCINE RESISTANT/IAA-LEUCINE RESISTANT-LIKE; IM, inflorescence meristem; IMA, INHIBITOR OF MERISTEM ACTIVITY; IQD, IQ67-domain protein; J, JOINTLESS; JA, jasmonate; KD, KN-like domain protein; KLUH, HULK backward; KNOX/KN, KNOTTED-LIKE; KNU, KNUCKLES; KPP, kinase partner protein; LAM, LAMINA; LC, LOCULE NUMBER; LIN, LONG INFLORESCENCE; LRR, leucine-rich repeat; LSD/LOL, LESION SIMULATING DISEASE/LESION SIMULATING DISEASE-LIKE; LST, LONG STYLES; MADS1, MADS-box transcription factor 1; MAP, microtubule-associated protein; MBP, MADS-box protein; MC, MACROCALYX; MED, mediator complex subunit; MET, methyltransferase; miR, microRNA; MPK, mitogen-activated protein kinase; MSH2, MutatorS-Homolog 2; MYB, myeloblastosis; NCED, 9-*cis*-epoxycarotenoid dioxygenase; ncRNA, non-coding RNA; OE, overexpression; OFP, OVATE family protein; P4H, proline 4-hydroxylase; PAD, PARENTAL ADVICE; PIF, phytochrome-interacting factor; PIN, PIN-FORMED; PLAC, PLACENTA ASSOCIATED; POD, POLLEN DEFICIENT; PP, protein phosphatase; PRP, proline-rich protein; PYR/PYL, PYRABACTIN RESISTANCE/PYRABACTIN RESISTANCE-LIKE; QTL, quantitative trait locus; RBOH, respiratory burst oxidase homolog; RIN, RIPENING INHIBITOR; RLK, receptor-like kinase; ROS, reactive oxygen species; SA, salicylate; SAM, shoot apical meristem; SB, SUPPRESSOR OF BRANCHING; SBP, SQUAMOSA promoter-binding protein; SEP, SEPALLATA; SES, SEXUAL STERILITY; SFT, SINGLE FLOWER TRUSS; SINA, SEVEN IN ABSENTIA; SLOMO, SLOW MOTION; SP, SELF-PRUNING; SPL, SPOROCYTELESS; SPMS, spermidine synthase; STM3, SISTER OF TM 3; STR, strictosidine synthase; SUC, sucrose; SWEET, SUGAR WILL EVENTUALLY BE EXPORTED TRANSPORTER; TAG/TAGL, tomato AGAMOUS/tomato AGAMOUS-like; TALE, three amino acid loop extension; TAPG, tomato abscission polygalacturonase; TDF, DEFECTIVE IN TAPETAL DEVELOPMENT AND FUNCTION; TFAM, TMF FAMILY MEMBER; TFL, TERMINAL FLOWER; TIP, tonoplast intrinsic protein; TIR, TRANSPORT INHIBITOR RESPONSE; TM, transition meristem; TM_, tomato MADS; TMF, TERMINATING FLOWER; TOE, TARGET OF EAT; TPD, TAPETUM DETERMINANT; TPL, TOPLESS; TRK, tomato receptor-like cytoplasmic kinase; TRM, TONNEAU1-recruiting motif; VM, vegetative meristem; WOX, WUS-related homeobox; WT, wild type; WUS, WUSCHEL; YUC, YUCCA; ZPR, LITTLE ZIPPER.

## References

[1] Molesini B., Dusi V., Pennisi F., Pandolfini T. (2020). How Hormones and MADS-Box Transcription Factors Are Involved in Controlling Fruit Set and Parthenocarpy in Tomato. Genes.

[2] Ezura K., Nomura Y., Ariizumi T. (2023). Molecular, Hormonal, and Metabolic Mechanisms of Fruit Set, the Ovary-to-Fruit Transition, in Horticultural Crops. J. Exp. Bot..

[3] Hoshikawa K., Pham D., Ezura H., Schafleitner R., Nakashima K. (2021). Genetic and Molecular Mechanisms Conferring Heat Stress Tolerance in Tomato Plants. Front. Plant Sci..

[4] Fenn M.A., Giovannoni J.J. (2021). Phytohormones in Fruit Development and Maturation. Plant J..

[5] Yang D., Wang Z., Huang X., Xu C. (2023). Molecular Regulation of Tomato Male Reproductive Development. Abiotech.

[6] Pandolfini T., Molesini B., Spena A. (2007). Molecular Dissection of the Role of Auxin in Fruit Initiation. Trends Plant Sci..

[7] Serrani J.C., Ruiz-Rivero O., Fos M., García-Martínez J.L. (2008). Auxin-induced Fruit-set in Tomato Is Mediated in Part by Gibberellins. Plant J..

[8] Llop-Tous I., Barry C.S., Grierson D. (2000). Regulation of Ethylene Biosynthesis in Response to Pollination in Tomato Flowers. Plant Physiol..

[9] de Jong M., Wolters-Arts M., García-Martínez J.L., Mariani C., Vriezen W.H. (2011). The *Solanum lycopersicum* AUXIN RESPONSE FACTOR 7 (SlARF7) Mediates Cross-Talk between Auxin and Gibberellin Signalling during Tomato Fruit Set and Development. J. Exp. Bot..

[10] Quinet M., Angosto T., Yuste-Lisbona F.J., Blanchard-Gros R., Bigot S., Martinez J.-P., Lutts S. (2019). Tomato Fruit Development and Metabolism. Front. Plant Sci..

[11] Gerszberg A., Hnatuszko-Konka K., Kowalczyk T., Kononowicz A.K. (2015). Tomato (*Solanum lycopersicum* L.) in the Service of Biotechnology. Plant Cell Tissue Organ Cult..

[12] Li Y., Chen Y., Zhou L., You S., Deng H., Chen Y., Alseekh S., Yuan Y., Fu R., Zhang Z. (2020). MicroTom Metabolic Network: Rewiring Tomato Metabolic Regulatory Network throughout the Growth Cycle. Mol. Plant.

[13] Bastías A., López-Climent M., Valcárcel M., Rosello S., Gómez-Cadenas A., Casaretto J.A. (2011). Modulation of Organic Acids and Sugar Content in Tomato Fruits by an Abscisic Acid-regulated Transcription Factor. Physiol. Plant..

[14] Li X., Wang Y., Chen S., Tian H., Fu D., Zhu B., Luo Y., Zhu H. (2018). Lycopene Is Enriched in Tomato Fruit by CRISPR/Cas9-Mediated Multiplex Genome Editing. Front. Plant Sci..

[15] Chen C., Zhang M., Zhang M., Yang M., Dai S., Meng Q., Lv W., Zhuang K. (2023). ETHYLENE-INSENSITIVE 3-LIKE 2 Regulates β-Carotene and Ascorbic Acid Accumulation in Tomatoes during Ripening. Plant Physiol..

[16] Ratto F., Franchini F., Musicco M., Caruso G., Di Santo S.G. (2022). A Narrative Review on the Potential of Tomato and Lycopene for the Prevention of Alzheimer’s Disease and Other Dementias. Crit. Rev. Food Sci. Nutr..

[17] Landrier J.-F., Breniere T., Sani L., Desmarchelier C., Mounien L., Borel P. (2023). Effect of Tomato, Tomato-Derived Products and Lycopene on Metabolic Inflammation: From Epidemiological Data to Molecular Mechanisms. Nutr. Res. Rev..

[18] Collins E.J., Bowyer C., Tsouza A., Chopra M. (2022). Tomatoes: An Extensive Review of the Associated Health Impacts of Tomatoes and Factors That Can Affect Their Cultivation. Biology.

[19] Rothan C., Diouf I., Causse M. (2019). Trait Discovery and Editing in Tomato. Plant J..

[20] Tiwari J.K., Singh A.K., Behera T.K. (2023). CRISPR/Cas Genome Editing in Tomato Improvement: Advances and Applications. Front. Plant Sci..

[21] Sussex I.M., Kerk N.M. (2001). The Evolution of Plant Architecture. Curr. Opin. Plant Biol..

[22] McGarry R.C., Ayre B.G. (2012). Manipulating Plant Architecture with Members of the CETS Gene Family. Plant Sci..

[23] Lippman Z.B., Cohen O., Alvarez J.P., Abu-Abied M., Pekker I., Paran I., Eshed Y., Zamir D. (2008). The Making of a Compound Inflorescence in Tomato and Related Nightshades. PLoS Biol..

[24] Molinero-Rosales N., Latorre A., Jamilena M., Lozano R. (2004). SINGLE FLOWER TRUSS Regulates the Transition and Maintenance of Flowering in Tomato. Planta.

[25] Robledo J.M., Medeiros D., Vicente M.H., Azevedo A.A., Thompson A.J., Peres L.E.P., Ribeiro D.M., Araújo W.L., Zsögön A. (2020). Control of Water-use Efficiency by Florigen. Plant Cell Environ..

[26] Park S.J., Jiang K., Tal L., Yichie Y., Gar O., Zamir D., Eshed Y., Lippman Z.B. (2014). Optimization of Crop Productivity in Tomato Using Induced Mutations in the Florigen Pathway. Nat. Genet..

[27] Shalit-Kaneh A., Eviatar-Ribak T., Horev G., Suss N., Aloni R., Eshed Y., Lifschitz E. (2019). The Flowering Hormone Florigen Accelerates Secondary Cell Wall Biogenesis to Harmonize Vascular Maturation with Reproductive Development. Proc. Natl. Acad. Sci. USA.

[28] Cui L., Zheng F., Wang J., Zhang C., Zhang D., Gao S., Zhang C., Ye J., Zhang Y., Ouyang B. (2022). The Tomato CONSTANS-LIKE Protein SlCOL1 Regulates Fruit Yield by Repressing SFT Gene Expression. BMC Plant Biol..

[29] Yang T., He Y., Niu S., Yan S., Zhang Y. (2020). Identification and Characterization of the CONSTANS (CO)/CONSTANS-like (COL) Genes Related to Photoperiodic Signaling and Flowering in Tomato. Plant Sci..

[30] Molesini B., Dusi V., Pennisi F., Di Sansebastiano G.P., Zanzoni S., Manara A., Furini A., Martini F., Rotino G.L., Pandolfini T. (2020). TCMP-2 Affects Tomato Flowering and Interacts with BBX16, a Homolog of the Arabidopsis B-box MiP1b. Plant Direct.

[31] Shibuya T., Nishiyama M., Kato K., Kanayama Y. (2021). Characterization of the FLAVIN-BINDING, KELCH REPEAT, F-BOX 1 Homolog SlFKF1 in Tomato as a Model for Plants with Fleshy Fruit. Int. J. Mol. Sci..

[32] Cui L., Zheng F., Wang J., Zhang C., Xiao F., Ye J., Li C., Ye Z., Zhang J. (2020). MiR156a-targeted SBP-Box Transcription Factor SlSPL13 Regulates Inflorescence Morphogenesis by Directly Activating *SFT* in Tomato. Plant Biotechnol. J..

[33] Silva G.F.F.E., Silva E.M., da Silva Azevedo M., Guivin M.A.C., Ramiro D.A., Figueiredo C.R., Carrer H., Peres L.E.P., Nogueira F.T.S. (2014). MicroRNA156-targeted SPL/SBP Box Transcription Factors Regulate Tomato Ovary and Fruit Development. Plant J..

[34] Silva G.F.F., Silva E.M., Correa J.P.O., Vicente M.H., Jiang N., Notini M.M., Junior A.C., De Jesus F.A., Castilho P., Carrera E. (2019). Tomato Floral Induction and Flower Development Are Orchestrated by the Interplay between Gibberellin and Two Unrelated MicroRNA-controlled Modules. New Phytol..

[35] Ferigolo L.F., Vicente M.H., Correa J.P.O., Barrera-Rojas C.H., Silva E.M., Silva G.F.F., Carvalho A., Peres L.E.P., Ambrosano G.B., Margarido G.R.A. (2023). Gibberellin and MiRNA156-Targeted SlSBP Genes Synergistically Regulate Tomato Floral Meristem Determinacy and Ovary Patterning. Development.

[36] Chen S., Song X., Zheng Q., Liu Y., Yu J., Zhou Y., Xia X. (2023). The Transcription Factor SPL13 Mediates Strigolactone Suppression of Shoot Branching by Inhibiting Cytokinin Synthesis in *Solanum lycopersicum*. J. Exp. Bot..

[37] Barrera-Rojas C.H., Vicente M.H., Pinheiro Brito D.A., Silva E.M., Lopez A.M., Ferigolo L.F., do Carmo R.M., Silva C.M.S., Silva G.F.F., Correa J.P.O. (2023). Tomato MiR156-Targeted *SlSBP15* Represses Shoot Branching by Modulating Hormone Dynamics and Interacting with *GOBLET* and *BRANCHED1b*. J. Exp. Bot..

[38] Zhou S., Hu Z., Li F., Yu X., Naeem M., Zhang Y., Chen G. (2018). Manipulation of Plant Architecture and Flowering Time by Down-Regulation of the GRAS Transcription Factor SlGRAS26 in *Solanum lycopersicum*. Plant Sci..

[39] Wu Y.-M., Ma Y.-J., Wang M., Zhou H., Gan Z.-M., Zeng R.-F., Ye L.-X., Zhou J.-J., Zhang J.-Z., Hu C.-G. (2022). Mobility of FLOWERING LOCUS T Protein as a Systemic Signal in Trifoliate Orange and Its Low Accumulation in Grafted Juvenile Scions. Hortic. Res..

[40] Kang M.-S., Kim Y.J., Heo J., Rajendran S., Wang X., Bae J.H., Lippman Z., Park S.J. (2022). Newly Discovered Alleles of the Tomato Antiflorigen Gene SELF PRUNING Provide a Range of Plant Compactness and Yield. Int. J. Mol. Sci..

[41] Cao K., Yan F., Xu D., Ai K., Yu J., Bao E., Zou Z. (2018). Phytochrome B1-Dependent Control of SP5G Transcription Is the Basis of the Night Break and Red to Far-Red Light Ratio Effects in Tomato Flowering. BMC Plant Biol..

[42] Soyk S., Müller N.A., Park S.J., Schmalenbach I., Jiang K., Hayama R., Zhang L., Van Eck J., Jiménez-Gómez J.M., Lippman Z.B. (2017). Variation in the Flowering Gene SELF PRUNING 5G Promotes Day-Neutrality and Early Yield in Tomato. Nat. Genet..

[43] Moreira J.D.R., Quiñones A., Lira B.S., Robledo J.M., Curtin S.J., Vicente M.H., Ribeiro D.M., Ryngajllo M., Jiménez-Gómez J.M., Peres L.E.P. (2022). *SELF PRUNING 3C* Is a Flowering Repressor That Modulates Seed Germination, Root Architecture, and Drought Responses. J. Exp. Bot..

[44] Hollwey E., Out S., Watson M.R., Heidmann I., Meyer P. (2017). *TET3*-Mediated Demethylation in Tomato Activates Expression of a *CETS* Gene That Stimulates Vegetative Growth. Plant Direct.

[45] Lifschitz E., Eviatar T., Rozman A., Shalit A., Goldshmidt A., Amsellem Z., Alvarez J.P., Eshed Y. (2006). The Tomato *FT* Ortholog Triggers Systemic Signals That Regulate Growth and Flowering and Substitute for Diverse Environmental Stimuli. Proc. Natl. Acad. Sci. USA.

[46] Shalit A., Rozman A., Goldshmidt A., Alvarez J.P., Bowman J.L., Eshed Y., Lifschitz E. (2009). The Flowering Hormone Florigen Functions as a General Systemic Regulator of Growth and Termination. Proc. Natl. Acad. Sci. USA.

[47] Pnueli L., Carmel-Goren L., Hareven D., Gutfinger T., Alvarez J., Ganal M., Zamir D., Lifschitz E. (1998). The *SELF-PRUNING* Gene of Tomato Regulates Vegetative to Reproductive Switching of Sympodial Meristems and Is the Ortholog of *CEN* and *TFL1*. Development.

[48] Allen K., Sussex I. (1996). Falsiflora and Anantha Control Early Stages of Floral Meristem Development in Tomato (*Lycopersicon esculentum* Mill.). Planta.

[49] Molinero-Rosales N., Jamilena M., Zurita S., Gómez P., Capel J., Lozano R. (1999). FALSIFLORA, the Tomato Orthologue of FLORICAULA and LEAFY, Controls Flowering Time and Floral Meristem Identity. Plant J..

[50] Zhang Y., Zhang B., Yang T., Zhang J., Liu B., Zhan X., Liang Y. (2020). The GAMYB-like Gene SlMYB33 Mediates Flowering and Pollen Development in Tomato. Hortic. Res..

[51] Huang X., Tang L., Yu Y., Dalrymple J., Lippman Z.B., Xu C. (2018). Control of Flowering and Inflorescence Architecture in Tomato by Synergistic Interactions between ALOG Transcription Factors. J. Genet. Genomics.

[52] Huang X., Xiao N., Zou Y., Xie Y., Tang L., Zhang Y., Yu Y., Li Y., Xu C. (2022). Heterotypic Transcriptional Condensates Formed by Prion-like Paralogous Proteins Canalize Flowering Transition in Tomato. Genome Biol..

[53] Huang X., Chen S., Li W., Tang L., Zhang Y., Yang N., Zou Y., Zhai X., Xiao N., Liu W. (2021). ROS Regulated Reversible Protein Phase Separation Synchronizes Plant Flowering. Nat. Chem. Biol..

[54] Izhaki A., Alvarez J.P., Cinnamon Y., Genin O., Liberman-Aloni R., Eyal Y. (2018). The Tomato BLADE ON PETIOLE and TERMINATING FLOWER Regulate Leaf Axil Patterning along the Proximal-Distal Axes. Front. Plant Sci..

[55] Xu C., Park S.J., Van Eck J., Lippman Z.B. (2016). Control of Inflorescence Architecture in Tomato by BTB/POZ Transcriptional Regulators. Genes Dev..

[56] Roldan M.V.G., Périlleux C., Morin H., Huerga-Fernandez S., Latrasse D., Benhamed M., Bendahmane A. (2017). Natural and Induced Loss of Function Mutations in SlMBP21 MADS-Box Gene Led to Jointless-2 Phenotype in Tomato. Sci. Rep..

[57] Liu D., Wang D., Qin Z., Zhang D., Yin L., Wu L., Colasanti J., Li A., Mao L. (2014). The SEPALLATA MADS-box Protein SLMBP21 Forms Protein Complexes with JOINTLESS and MACROCALYX as a Transcription Activator for Development of the Tomato Flower Abscission Zone. Plant J..

[58] Li N., Huang B., Tang N., Jian W., Zou J., Chen J., Cao H., Habib S., Dong X., Wei W. (2017). The MADS-Box Gene SlMBP21 Regulates Sepal Size Mediated by Ethylene and Auxin in Tomato. Plant Cell Physiol..

[59] Zhang D., Ai G., Ji K., Huang R., Chen C., Yang Z., Wang J., Cui L., Li G., Tahira M. (2023). *EARLY FLOWERING* Is a Dominant Gain-of-function Allele of *FANTASTIC FOUR 1/2c* That Promotes Early Flowering in Tomato. Plant Biotechnol. J..

[60] Soyk S., Lemmon Z.H., Oved M., Fisher J., Liberatore K.L., Park S.J., Goren A., Jiang K., Ramos A., van der Knaap E. (2017). Bypassing Negative Epistasis on Yield in Tomato Imposed by a Domestication Gene. Cell.

[61] Zhang J., Hu Z., Wang Y., Yu X., Liao C., Zhu M., Chen G. (2018). Suppression of a Tomato SEPALLATA MADS-Box Gene, SlCMB1, Generates Altered Inflorescence Architecture and Enlarged Sepals. Plant Sci..

[62] Soyk S., Lemmon Z.H., Sedlazeck F.J., Jiménez-Gómez J.M., Alonge M., Hutton S.F., Van Eck J., Schatz M.C., Lippman Z.B. (2019). Duplication of a Domestication Locus Neutralized a Cryptic Variant That Caused a Breeding Barrier in Tomato. Nat. Plants.

[63] Alonge M., Wang X., Benoit M., Soyk S., Pereira L., Zhang L., Suresh H., Ramakrishnan S., Maumus F., Ciren D. (2020). Major Impacts of Widespread Structural Variation on Gene Expression and Crop Improvement in Tomato. Cell.

[64] Wang X., Liu Z., Bai J., Sun S., Song J., Li R., Cui X. (2023). Antagonistic Regulation of Target Genes by the SISTER OF TM3–JOINTLESS2 Complex in Tomato Inflorescence Branching. Plant Cell.

[65] Wang X., Liu Z., Sun S., Wu J., Li R., Wang H., Cui X. (2021). SISTER OF TM3 Activates FRUITFULL1 to Regulate Inflorescence Branching in Tomato. Hortic. Res..

[66] Sun S., Wang X., Liu Z., Bai J., Song J., Li R., Cui X. (2023). Tomato APETALA2 Family Member SlTOE1 Regulates Inflorescence Branching by Repressing *SISTER OF TM3*. Plant Physiol..

[67] Maheepala D.C., Emerling C.A., Rajewski A., Macon J., Strahl M., Pabón-Mora N., Litt A. (2019). Evolution and Diversification of FRUITFULL Genes in Solanaceae. Front. Plant Sci..

[68] Jiang X., Lubini G., Hernandes-Lopes J., Rijnsburger K., Veltkamp V., de Maagd R.A., Angenent G.C., Bemer M. (2022). FRUITFULL-like Genes Regulate Flowering Time and Inflorescence Architecture in Tomato. Plant Cell.

[69] Zahn I.E., Roelofsen C., Angenent G.C., Bemer M. (2023). TM3 and STM3 Promote Flowering Together with FUL2 and MBP20, but Act Antagonistically in Inflorescence Branching in Tomato. Plants.

[70] Baranov D., Timerbaev V. (2024). Recent Advances in Studying the Regulation of Fruit Ripening in Tomato Using Genetic Engineering Approaches. Int. J. Mol. Sci..

[71] Yuste-Lisbona F.J., Quinet M., Fernández-Lozano A., Pineda B., Moreno V., Angosto T., Lozano R. (2016). Characterization of Vegetative Inflorescence (Mc-Vin) Mutant Provides New Insight into the Role of MACROCALYX in Regulating Inflorescence Development of Tomato. Sci. Rep..

[72] Xing M., Li H., Liu G., Zhu B., Zhu H., Grierson D., Luo Y., Fu D. (2022). A MADS-Box Transcription Factor, SlMADS1, Interacts with SlMACROCALYX to Regulate Tomato Sepal Growth. Plant Sci..

[73] Li F., Jia Y., Zhou S., Chen X., Xie Q., Hu Z., Chen G. (2022). SlMBP22 Overexpression in Tomato Affects Flower Morphology and Fruit Development. J. Plant Physiol..

[74] Yu X., Chen G., Guo X., Lu Y., Zhang J., Hu J., Tian S., Hu Z. (2017). Silencing SlAGL6, a Tomato AGAMOUS-LIKE6 Lineage Gene, Generates Fused Sepal and Green Petal. Plant Cell Rep..

[75] Gimenez E., Castañeda L., Pineda B., Pan I.L., Moreno V., Angosto T., Lozano R. (2016). TOMATO AGAMOUS1 and ARLEQUIN/TOMATO AGAMOUS-LIKE1 MADS-Box Genes Have Redundant and Divergent Functions Required for Tomato Reproductive Development. Plant Mol. Biol..

[76] Nezhdanova A.V., Slugina M.A., Dyachenko E.A., Kamionskaya A.M., Kochieva E.Z., Shchennikova A.V. (2021). Analysis of the Structure and Function of the Tomato *Solanum Lycopersicum* L. MADS-Box Gene *SlMADS5*. Vavilovskii Zh. Genet. Selektsii.

[77] Slugina M.A., Dyachenko E.A., Kochieva E.Z., Shchennikova A.V. (2020). Structural and Functional Diversification of SEPALLATA Genes TM5 and RIN in Tomato Species (*Section lycopersicon*). Dokl. Biochem. Biophys..

[78] Ampomah-Dwamena C., Morris B.A., Sutherland P., Veit B., Yao J.-L. (2002). Down-Regulation of *TM29*, a Tomato *SEPALLATA* Homolog, Causes Parthenocarpic Fruit Development and Floral Reversion. Plant Physiol..

[79] Zhao L., Lu J., Zhang J., Wu P.-Y., Yang S., Wu K. (2015). Identification and Characterization of Histone Deacetylases in Tomato (*Solanum lycopersicum*). Front. Plant Sci..

[80] Arhondakis S., Bita C.E., Perrakis A., Manioudaki M.E., Krokida A., Kaloudas D., Kalaitzis P. (2016). In Silico Transcriptional Regulatory Networks Involved in Tomato Fruit Ripening. Front. Plant Sci..

[81] Rojas-Gracia P., Roque E., Medina M., López-Martín M.J., Cañas L.A., Beltrán J.P., Gómez-Mena C. (2019). The DOF Transcription Factor SlDOF10 Regulates Vascular Tissue Formation during Ovary Development in Tomato. Front. Plant Sci..

[82] Hu G., Wang K., Huang B., Mila I., Frasse P., Maza E., Djari A., Hernould M., Zouine M., Li Z. (2022). The Auxin-Responsive Transcription Factor SlDOF9 Regulates Inflorescence and Flower Development in Tomato. Nat. Plants.

[83] Ewas M., Khames E., Ziaf K., Shahzad R., Nishawy E., Ali F., Subthain H., Amar M.H., Ayaad M., Ghaly O. (2017). The Tomato DOF Daily Fluctuations 1, TDDF1 Acts as Flowering Accelerator and Protector against Various Stresses. Sci. Rep..

[84] Xu D., Li X., Wu X., Meng L., Zou Z., Bao E., Bian Z., Cao K. (2021). Tomato SlCDF3 Delays Flowering Time by Regulating Different FT-like Genes under Long-Day and Short-Day Conditions. Front. Plant Sci..

[85] Yang L., Qi S., Touqeer A., Li H., Zhang X., Liu X., Wu S. (2020). SlGT11 Controls Floral Organ Patterning and Floral Determinacy in Tomato. BMC Plant Biol..

[86] Bertin N. (2004). Analysis of the Tomato Fruit Growth Response to Temperature and Plant Fruit Load in Relation to Cell Division, Cell Expansion and DNA Endoreduplication. Ann. Bot..

[87] Taylor J.E., Whitelaw C.A. (2001). Signals in Abscission. New Phytol..

[88] Butenko M.A., Simon R. (2015). Beyond the Meristems: Similarities in the CLAVATA3 and INFLORESCENCE DEFICIENT IN ABSCISSION Peptide Mediated Signalling Pathways. J. Exp. Bot..

[89] Jiang C., Liang Y., Deng S., Liu Y., Zhao H., Li S., Jiang C.-Z., Gao J., Ma C. (2023). The *Rh*LOL1–*Rh*ILR3 Module Mediates Cytokinin-induced Petal Abscission in Rose. New Phytol..

[90] Li R., Shi C.-L., Wang X., Meng Y., Cheng L., Jiang C.-Z., Qi M., Xu T., Li T. (2021). Inflorescence Abscission Protein SlIDL6 Promotes Low Light Intensity-Induced Tomato Flower Abscission. Plant Physiol..

[91] Zhao M., Li C., Ma X., Xia R., Chen J., Liu X., Ying P., Peng M., Wang J., Shi C.-L. (2020). KNOX Protein KNAT1 Regulates Fruitlet Abscission in Litchi by Repressing Ethylene Biosynthetic Genes. J. Exp. Bot..

[92] Fu X., Shi Z., Jiang Y., Jiang L., Qi M., Xu T., Li T. (2019). A Family of Auxin Conjugate Hydrolases from *Solanum lycopersicum* and Analysis of Their Roles in Flower Pedicel Abscission. BMC Plant Biol..

[93] Yan F., Gong Z., Hu G., Ma X., Bai R., Yu R., Zhang Q., Deng W., Li Z., Wuriyanghan H. (2021). Tomato SlBL4 Plays an Important Role in Fruit Pedicel Organogenesis and Abscission. Hortic. Res..

[94] Shi Z., Jiang Y., Han X., Liu X., Cao R., Qi M., Xu T., Li T. (2017). SlPIN1 Regulates Auxin Efflux to Affect Flower Abscission Process. Sci. Rep..

[95] Li A., Chen G., Yu X., Zhu Z., Zhang L., Zhou S., Hu Z. (2019). The Tomato MADS-Box Gene SlMBP9 Negatively Regulates Lateral Root Formation and Apical Dominance by Reducing Auxin Biosynthesis and Transport. Plant Cell Rep..

[96] Wang R., Li R., Cheng L., Wang X., Fu X., Dong X., Qi M., Jiang C., Xu T., Li T. (2021). SlERF52 Regulates *SlTIP1;1* Expression to Accelerate Tomato Pedicel Abscission. Plant Physiol..

[97] Sundaresan S., Philosoph-Hadas S., Ma C., Jiang C.-Z., Riov J., Mugasimangalam R., Kochanek B., Salim S., Reid M.S., Meir S. (2018). The Tomato Hybrid Proline-Rich Protein Regulates the Abscission Zone Competence to Respond to Ethylene Signals. Hortic. Res..

[98] Perrakis A., Bita C.E., Arhondakis S., Krokida A., Mekkaoui K., Denic D., Blazakis K.N., Kaloudas D., Kalaitzis P. (2019). Suppression of a Prolyl 4 Hydroxylase Results in Delayed Abscission of Overripe Tomato Fruits. Front. Plant Sci..

[99] Qi X., Hu S., Zhou H., Liu X., Wang L., Zhao B., Huang X., Zhang S. (2018). A MADS-Box Transcription Factor of ‘Kuerlexiangli’(Pyrus Sinkiangensis Yu) PsJOINTLESS Gene Functions in Floral Organ Abscission. Gene.

[100] Deng H., Pirrello J., Chen Y., Li N., Zhu S., Chirinos X., Bouzayen M., Liu Y., Liu M. (2018). A Novel Tomato F-box Protein, SlEBF3, Is Involved in Tuning Ethylene Signaling during Plant Development and Climacteric Fruit Ripening. Plant J..

[101] Zhang Y., Li Q., Jiang L., Kai W., Liang B., Wang J., Du Y., Zhai X., Wang J., Zhang Y. (2018). Suppressing Type 2C Protein Phosphatases Alters Fruit Ripening and the Stress Response in Tomato. Plant Cell Physiol..

[102] Kai W., Wang J., Liang B., Fu Y., Zheng Y., Zhang W., Li Q., Leng P. (2019). PYL9 Is Involved in the Regulation of ABA Signaling during Tomato Fruit Ripening. J. Exp. Bot..

[103] Kai W., Fu Y., Wang J., Liang B., Li Q., Leng P. (2019). Functional Analysis of SlNCED1 in Pistil Development and Fruit Set in Tomato (*Solanum lycopersicum* L.). Sci. Rep..

[104] Wang R., Shi C., Wang X., Li R., Meng Y., Cheng L., Qi M., Xu T., Li T. (2020). Tomato SlIDA Has a Critical Role in Tomato Fertilization by Modifying Reactive Oxygen Species Homeostasis. Plant J..

[105] Sundaresan S., Philosoph-Hadas S., Ma C., Jiang C.-Z., Riov J., Kochanek B., Salim S., Reid M.S., Meir S. (2021). Role of the KNOTTED1-LIKE HOMEOBOX Protein (KD1) in Regulating Abscission of Tomato Flower Pedicels at Early and Late Stages of the Process. Physiol. Plant..

[106] Kaulfürst-Soboll H., Mertens-Beer M., Brehler R., Albert M., von Schaewen A. (2021). Complex N-Glycans Are Important for Normal Fruit Ripening and Seed Development in Tomato. Front. Plant Sci..

[107] Wang X., Meng J., Deng L., Wang Y., Liu H., Yao J.-L., Nieuwenhuizen N.J., Wang Z., Zeng W. (2021). Diverse Functions of IAA-Leucine Resistant PpILR1 Provide a Genic Basis for Auxin-Ethylene Crosstalk during Peach Fruit Ripening. Front. Plant Sci..

[108] Cheng L., Li R., Wang X., Ge S., Wang S., Liu X., He J., Jiang C.-Z., Qi M., Xu T. (2022). A SlCLV3-*SlWUS* Module Regulates Auxin and Ethylene Homeostasis in Low Light-Induced Tomato Flower Abscission. Plant Cell.

[109] Liu X., Cheng L., Li R., Cai Y., Wang X., Fu X., Dong X., Qi M., Jiang C.-Z., Xu T. (2022). The HD-Zip Transcription Factor SlHB15A Regulates Abscission by Modulating Jasmonoyl-Isoleucine Biosynthesis. Plant Physiol..

[110] Meng S., Xiang H., Yang X., Ye Y., Han L., Xu T., Liu Y., Wang F., Tan C., Qi M. (2023). Effects of Low Temperature on Pedicel Abscission and Auxin Synthesis Key Genes of Tomato. Int. J. Mol. Sci..

[111] Li J., Liu Y., Zhang J., Cao L., Xie Q., Chen G., Chen X., Hu Z. (2023). Suppression of a Hexokinase Gene SlHXK1 in Tomato Affects Fruit Setting and Seed Quality. Plant Physiol. Biochem..

[112] Chu Y.-H., Jang J.-C., Huang Z., van der Knaap E. (2019). Tomato Locule Number and Fruit Size Controlled by Natural Alleles of *Lc* and *Fas*. Plant Direct.

[113] Cong B., Barrero L.S., Tanksley S.D. (2008). Regulatory Change in YABBY-like Transcription Factor Led to Evolution of Extreme Fruit Size during Tomato Domestication. Nat. Genet..

[114] Xu C., Liberatore K.L., MacAlister C.A., Huang Z., Chu Y.-H., Jiang K., Brooks C., Ogawa-Ohnishi M., Xiong G., Pauly M. (2015). A Cascade of Arabinosyltransferases Controls Shoot Meristem Size in Tomato. Nat. Genet..

[115] Sun M., Li H., Li Y., Xiang H., Liu Y., He Y., Qi M., Li T. (2020). Tomato YABBY2b Controls Plant Height through Regulating Indole-3-Acetic Acid-Amido Synthetase (GH3.8) Expression. Plant Sci..

[116] Li H., Qi M., Sun M., Liu Y., Liu Y., Xu T., Li Y., Li T. (2017). Tomato Transcription Factor SlWUS Plays an Important Role in Tomato Flower and Locule Development. Front. Plant Sci..

[117] Hendelman A., Zebell S., Rodriguez-Leal D., Dukler N., Robitaille G., Wu X., Kostyun J., Tal L., Wang P., Bartlett M.E. (2021). Conserved Pleiotropy of an Ancient Plant Homeobox Gene Uncovered by *Cis*-Regulatory Dissection. Cell.

[118] Zhang C., Wang J., Wang X., Li C., Ye Z., Zhang J. (2020). UF, a WOX Gene, Regulates a Novel Phenotype of Un-Fused Flower in Tomato. Plant Sci..

[119] Wang C., Zhao B., He L., Zhou S., Liu Y., Zhao W., Guo S., Wang R., Bai Q., Li Y. (2021). The WOX Family Transcriptional Regulator SlLAM1 Controls Compound Leaf and Floral Organ Development in *Solanum lycopersicum*. J. Exp. Bot..

[120] Du F., Mo Y., Israeli A., Wang Q., Yifhar T., Ori N., Jiao Y. (2020). Leaflet Initiation and Blade Expansion Are Separable in Compound Leaf Development. Plant J..

[121] Su D., Wen L., Xiang W., Shi Y., Lu W., Liu Y., Xian Z., Li Z. (2022). Tomato Transcriptional Repressor SlBES1.8 Influences Shoot Apical Meristem Development by Inhibiting the DNA Binding Ability of SlWUS. Plant J..

[122] Sun B., Zhou Y., Cai J., Shang E., Yamaguchi N., Xiao J., Looi L.-S., Wee W.-Y., Gao X., Wagner D. (2019). Integration of Transcriptional Repression and Polycomb-Mediated Silencing of *WUSCHEL* in Floral Meristems. Plant Cell.

[123] Bollier N., Sicard A., Leblond J., Latrasse D., Gonzalez N., Gévaudant F., Benhamed M., Raynaud C., Lenhard M., Chevalier C. (2018). At-MINI ZINC FINGER2 and Sl-INHIBITOR OF MERISTEM ACTIVITY, a Conserved Missing Link in the Regulation of Floral Meristem Termination in Arabidopsis and Tomato. Plant Cell.

[124] Bollier N., Sicard A., Gonzalez N., Chevalier C., Hernould M., Delmas F. (2018). Induced Ovule-to-Flower Switch by Interfering with SlIMA Activity in Tomato. Plant Signal. Behav..

[125] Castañeda L., Giménez E., Pineda B., García-Sogo B., Ortiz-Atienza A., Micol-Ponce R., Angosto T., Capel J., Moreno V., Yuste-Lisbona F.J. (2022). Tomato *CRABS CLAW* Paralogues Interact with Chromatin Remodelling Factors to Mediate Carpel Development and Floral Determinacy. New Phytol..

[126] Xu Q., Li R., Weng L., Sun Y., Li M., Xiao H. (2019). Domain-Specific Expression of Meristematic Genes Is Defined by the LITTLE ZIPPER Protein DTM in Tomato. Commun. Biol..

[127] Jaiswal N., Liao C.-J., Mengesha B., Han H., Lee S., Sharon A., Zhou Y., Mengiste T. (2022). Regulation of Plant Immunity and Growth by Tomato Receptor-like Cytoplasmic Kinase TRK1. New Phytol..

[128] Song S., Huang B., Pan Z., Zhong Q., Yang Y., Chen D., Zhu L., Hu G., He M., Wu C. (2022). The SlTPL3–SlWUS Module Regulates Multi-locule Formation in Tomato by Modulating Auxin and Gibberellin Levels in the Shoot Apical Meristem. J. Integr. Plant Biol..

[129] Yuste-Lisbona F.J., Fernández-Lozano A., Pineda B., Bretones S., Ortíz-Atienza A., García-Sogo B., Müller N.A., Angosto T., Capel J., Moreno V. (2020). *ENO* Regulates Tomato Fruit Size through the Floral Meristem Development Network. Proc. Natl. Acad. Sci. USA.

[130] Wang R., Tavano E.C.D.R., Lammers M., Martinelli A.P., Angenent G.C., de Maagd R.A. (2019). Re-Evaluation of Transcription Factor Function in Tomato Fruit Development and Ripening with CRISPR/Cas9-Mutagenesis. Sci. Rep..

[131] Ikeda M., Mitsuda N., Ohme-Takagi M. (2009). *Arabidopsis* WUSCHEL Is a Bifunctional Transcription Factor That Acts as a Repressor in Stem Cell Regulation and as an Activator in Floral Patterning. Plant Cell.

[132] Perales M., Rodriguez K., Snipes S., Yadav R.K., Diaz-Mendoza M., Reddy G.V. (2016). Threshold-Dependent Transcriptional Discrimination Underlies Stem Cell Homeostasis. Proc. Natl. Acad. Sci. USA.

[133] Hu C., Zhu Y., Cui Y., Cheng K., Liang W., Wei Z., Zhu M., Yin H., Zeng L., Xiao Y. (2018). A Group of Receptor Kinases Are Essential for CLAVATA Signalling to Maintain Stem Cell Homeostasis. Nat. Plants.

[134] Schoof H., Lenhard M., Haecker A., Mayer K.F.X., Jürgens G., Laux T. (2000). The Stem Cell Population of Arabidopsis Shoot Meristems Is Maintained by a Regulatory Loop between the CLAVATA and WUSCHEL Genes. Cell.

[135] Wang H., Tang X., Liu Y. (2023). SlCK2α as a Novel Substrate for CRL4 E3 Ligase Regulates Fruit Size through Maintenance of Cell Division Homeostasis in Tomato. Planta.

[136] Rodríguez G.R., Muños S., Anderson C., Sim S.-C., Michel A., Causse M., Gardener B.B.M., Francis D., van der Knaap E. (2011). Distribution of *SUN, OVATE, LC*, and *FAS* in the Tomato Germplasm and the Relationship to Fruit Shape Diversity. Plant Physiol..

[137] Liu J., Van Eck J., Cong B., Tanksley S.D. (2002). A New Class of Regulatory Genes Underlying the Cause of Pear-Shaped Tomato Fruit. Proc. Natl. Acad. Sci. USA.

[138] Rodríguez G.R., Kim H.J., van der Knaap E. (2013). Mapping of Two Suppressors of OVATE (Sov) Loci in Tomato. Heredity.

[139] Wang S., Chang Y., Ellis B. (2016). Overview of OVATE FAMILY PROTEINS, A Novel Class of Plant-Specific Growth Regulators. Front. Plant Sci..

[140] Chen J., Pan B., Li Z., Xu Y., Cao X., Jia J., Shen H., Sun L. (2023). Fruit Shape Loci Sun, Ovate, Fs8.1 and Their Interactions Affect Seed Size and Shape in Tomato. Front. Plant Sci..

[141] Liu J., Zhang J., Miao H., Jia C., Wang J., Xu B., Jin Z. (2017). Elucidating the Mechanisms of the Tomato *Ovate* Mutation in Regulating Fruit Quality Using Proteomics Analysis. J. Agric. Food Chem..

[142] Liu J., Zhang J., Wang J., Zhang J., Miao H., Jia C., Wang Z., Xu B., Jin Z. (2018). *MuMADS1* and *MaOFP1* Regulate Fruit Quality in a Tomato *Ovate* Mutant. Plant Biotechnol. J..

[143] Wu Q., Sun J., Fu J., Yu H., Wang X., Wang S., Adhikari P.B., Deng X., Xu Q. (2022). Genome-Wide Identification of Ovate Family in Citrus and Functional Characterization of CitOFP19. Plant Sci..

[144] Feng Z., Wu X., Wang J., Wu X., Wang B., Lu Z., Ye Z., Li G., Wang Y. (2022). Identification of Bottle Gourd (*Lagenaria siceraria*) OVATE Family Genes and Functional Characterization of LsOVATE1. Biomolecules.

[145] Zhou S., Cheng X., Li F., Feng P., Hu G., Chen G., Xie Q., Hu Z. (2019). Overexpression of SlOFP20 in Tomato Affects Plant Growth, Chlorophyll Accumulation, and Leaf Senescence. Front. Plant Sci..

[146] Zhou S., Hu Z., Li F., Tian S., Zhu Z., Li A., Chen G. (2019). Overexpression of SlOFP20 Affects Floral Organ and Pollen Development. Hortic. Res..

[147] Wu S., Xiao H., Cabrera A., Meulia T., van der Knaap E. (2011). *SUN* Regulates Vegetative and Reproductive Organ Shape by Changing Cell Division Patterns. Plant Physiol..

[148] Zhang B., Li Q., Keyhaninejad N., Taitano N., Sapkota M., Snouffer A., van der Knaap E. (2023). A Combinatorial TRM-OFP Module Bilaterally Fine-tunes Tomato Fruit Shape. New Phytol..

[149] Wu S., Zhang B., Keyhaninejad N., Rodríguez G.R., Kim H.J., Chakrabarti M., Illa-Berenguer E., Taitano N.K., Gonzalo M.J., Díaz A. (2018). A Common Genetic Mechanism Underlies Morphological Diversity in Fruits and Other Plant Organs. Nat. Commun..

[150] Wang Y., Clevenger J.P., Illa-Berenguer E., Meulia T., van der Knaap E., Sun L. (2019). A Comparison of Sun, Ovate, Fs8.1 and Auxin Application on Tomato Fruit Shape and Gene Expression. Plant Cell Physiol..

[151] Zheng H., Dong Y., Nong H., Huang L., Liu J., Yu X., Zhang Y., Yang L., Hong B., Wang W. (2022). *VvSUN* May Act in the Auxin Pathway to Regulate Fruit Shape in Grape. Hortic. Res..

[152] Bi L., Weng L., Jiang Z., Xiao H. (2018). The Tomato IQD Gene SUN24 Regulates Seed Germination through ABA Signaling Pathway. Planta.

[153] Dou J., Duan S., Umer M.J., Xie K., Wang Y., Kang Q., Yang S., Yang L., Liu D., Liu L. (2022). Genome-Wide Analysis of IQD Proteins and Ectopic Expression of Watermelon ClIQD24 in Tomato Suggests Its Important Role in Regulating Fruit Shape. Front. Genet..

[154] Bao Z., Guo Y., Deng Y., Zang J., Zhang J., Deng Y., Ouyang B., Qu X., Bürstenbinder K., Wang P. (2023). Microtubule-Associated Protein SlMAP70 Interacts with IQ67-Domain Protein SlIQD21a to Regulate Fruit Shape in Tomato. Plant Cell.

[155] Lazzaro M.D., Wu S., Snouffer A., Wang Y., van der Knaap E. (2018). Plant Organ Shapes Are Regulated by Protein Interactions and Associations with Microtubules. Front. Plant Sci..

[156] Shtern A., Keren-Keiserman A., Mauxion J.-P., Furumizu C., Alvarez J.P., Amsellem Z., Gil N., Motenko E., Alkalai-Tuvia S., Fallik E. (2023). *Solanum lycopersicum CLASS-II KNOX* Genes Regulate Fruit Anatomy via Gibberellin-Dependent and Independent Pathways. J. Exp. Bot..

[157] Keren-Keiserman A., Shtern A., Levy M., Chalupowicz D., Furumizu C., Alvarez J.P., Amsalem Z., Arazi T., Alkalai-Tuvia S., Efroni I. (2022). *CLASS-II KNOX* Genes Coordinate Spatial and Temporal Ripening in Tomato. Plant Physiol..

[158] Yao J., Zhang S., Wu N., Li X., Ahmad B., Wu J., Guo R., Wang X. (2023). KNOX Transcription Factor VvHB63 Affects Grape Seed Development by Interacting with Protein VvHB06. Plant Sci..

[159] Yan F., Deng W., Pang X., Gao Y., Chan H., Zhang Q., Hu N., Chen J., Li Z. (2019). Overexpression of the KNOX Gene Tkn4 Affects Pollen Development and Confers Sensitivity to Gibberellin and Auxin in Tomato. Plant Sci..

[160] Yan F., Gao Y., Pang X., Xu X., Zhu N., Chan H., Hu G., Wu M., Yuan Y., Li H. (2020). BEL1-LIKE HOMEODOMAIN4 Regulates Chlorophyll Accumulation, Chloroplast Development, and Cell Wall Metabolism in Tomato Fruit. J. Exp. Bot..

[161] Meng L., Fan Z., Zhang Q., Wang C., Gao Y., Deng Y., Zhu B., Zhu H., Chen J., Shan W. (2018). *BEL1-LIKE HOMEODOMAIN 11* Regulates Chloroplast Development and Chlorophyll Synthesis in Tomato Fruit. Plant J..

[162] Ezura K., Nakamura A., Mitsuda N. (2022). Genome-Wide Characterization of the TALE Homeodomain Family and the KNOX-BLH Interaction Network in Tomato. Plant Mol. Biol..

[163] Wang J., Zhao P., Cheng B., Zhang Y., Shen Y., Wang X., Zhang Q., Lou Q., Zhang S., Wang B. (2022). Identification of TALE Transcription Factor Family and Expression Patterns Related to Fruit Chloroplast Development in Tomato (*Solanum lycopersicum* L.). Int. J. Mol. Sci..

[164] He Y., Yang T., Yan S., Niu S., Zhang Y. (2022). Identification and Characterization of the BEL1-like Genes Reveal Their Potential Roles in Plant Growth and Abiotic Stress Response in Tomato. Int. J. Biol. Macromol..

[165] Li Q., Feng Q., Snouffer A., Zhang B., Rodríguez G.R., van der Knaap E. (2022). Increasing Fruit Weight by Editing a *Cis*-Regulatory Element in Tomato KLUH Promoter Using CRISPR/Cas9. Front. Plant Sci..

[166] Grandillo S., Ku H.M., Tanksley S.D. (1999). Identifying the Loci Responsible for Natural Variation in Fruit Size and Shape in Tomato. Züchter Genet. Breed. Res..

[167] Thibivilliers S., Farmer A., Libault M. (2020). Biological and Cellular Functions of the Microdomain-Associated FWL/CNR Protein Family in Plants. Plants.

[168] Beauchet A., Gévaudant F., Gonzalez N., Chevalier C. (2021). In Search of the Still Unknown Function of FW2.2/CELL NUMBER REGULATOR, a Major Regulator of Fruit Size in Tomato. J. Exp. Bot..

[169] Mu Q., Huang Z., Chakrabarti M., Illa-Berenguer E., Liu X., Wang Y., Ramos A., van der Knaap E. (2017). Fruit Weight Is Controlled by Cell Size Regulator Encoding a Novel Protein That Is Expressed in Maturing Tomato Fruits. PLoS Genet..

[170] Wu S., Clevenger J.P., Sun L., Visa S., Kamiya Y., Jikumaru Y., Blakeslee J., van der Knaap E. (2015). The Control of Tomato Fruit Elongation Orchestrated by Sun, Ovate and Fs8.1 in a Wild Relative of Tomato. Plant Sci..

[171] Sun L., Rodriguez G.R., Clevenger J.P., Illa-Berenguer E., Lin J., Blakeslee J.J., Liu W., Fei Z., Wijeratne A., Meulia T. (2015). Candidate Gene Selection and Detailed Morphological Evaluations of *Fs8.1*, a Quantitative Trait Locus Controlling Tomato Fruit Shape. J. Exp. Bot..

[172] Chaudhury A.M. (1993). Nuclear Genes Controlling Male Fertility. Plant Cell.

[173] McNeil K.J., Smith A.G. (2010). A Glycine-Rich Protein That Facilitates Exine Formation during Tomato Pollen Development. Planta.

[174] Jaffri S.R.F., Scheer H., MacAlister C.A. (2023). The Hydroxyproline O-Arabinosyltransferase FIN4 Is Required for Tomato Pollen Intine Development. Plant Reprod..

[175] Ochoa-Jiménez V.-A., Berumen-Varela G., Burgara-Estrella A., Orozco-Avitia J.-A., Ojeda-Contreras Á.-J., Trillo-Hernández E.-A., Rivera-Domínguez M., Troncoso-Rojas R., Báez-Sañudo R., Datsenka T. (2018). Functional Analysis of Tomato Rhamnogalacturonan Lyase Gene Solyc11g011300 during Fruit Development and Ripening. J. Plant Physiol..

[176] Jaffri S.R.F., MacAlister C.A. (2021). Sequential Deposition and Remodeling of Cell Wall Polymers during Tomato Pollen Development. Front. Plant Sci..

[177] Mascarenhas J.P. (1993). Molecular Mechanisms of Pollen Tube Growth and Differentiation. Plant Cell.

[178] Gillaspy G., Ben-David H., Gruissem W. (1993). Fruits: A Developmental Perspective. Plant Cell.

[179] Wu M., Zhang Q., Wu G., Zhang L., Xu X., Hu X., Gong Z., Chen Y., Li Z., Li H. (2023). SlMYB72 Affects Pollen Development by Regulating Autophagy in Tomato. Hortic. Res..

[180] Wu C., Yang Y., Su D., Yu C., Xian Z., Pan Z., Guan H., Hu G., Chen D., Li Z. (2022). The SlHB8 Acts as a Negative Regulator in Tapetum Development and Pollen Wall Formation in Tomato. Hortic. Res..

[181] Jung Y.J., Kim D.H., Lee H.J., Nam K.H., Bae S., Nou I.S., Cho Y.-G., Kim M.K., Kang K.K. (2020). Knockout of SlMS10 Gene (Solyc02g079810) Encoding BHLH Transcription Factor Using CRISPR/Cas9 System Confers Male Sterility Phenotype in Tomato. Plants.

[182] Liu X., Yang M., Liu X., Wei K., Cao X., Wang X., Wang X., Guo Y., Du Y., Li J. (2019). A Putative BHLH Transcription Factor Is a Candidate Gene for Male Sterile 32, a Locus Affecting Pollen and Tapetum Development in Tomato. Hortic. Res..

[183] Wang W., Fan Y., Niu X., Miao M., Kud J., Zhou B., Zeng L., Liu Y., Xiao F. (2018). Functional Analysis of the Seven in Absentia Ubiquitin Ligase Family in Tomato. Plant Cell Environ..

[184] Bao H., Ding Y., Yang F., Zhang J., Xie J., Zhao C., Du K., Zeng Y., Zhao K., Li Z. (2022). Gene Silencing, Knockout and over-Expression of a Transcription Factor ABORTED MICROSPORES (SlAMS) Strongly Affects Pollen Viability in Tomato (*Solanum lycopersicum*). BMC Genom..

[185] Du M., Zhou K., Liu Y., Deng L., Zhang X., Lin L., Zhou M., Zhao W., Wen C., Xing J. (2020). A Biotechnology-based Male-sterility System for Hybrid Seed Production in Tomato. Plant J..

[186] Chen L., Yang D., Zhang Y., Wu L., Zhang Y., Ye L., Pan C., He Y., Huang L., Ruan Y.-L. (2018). Evidence for a Specific and Critical Role of Mitogen-activated Protein Kinase 20 in Uni-to-binucleate Transition of Microgametogenesis in Tomato. New Phytol..

[187] Wang J., Li M., Zhuo S., Liu Y., Yu X., Mukhtar S., Ali M., Lu G. (2022). Mitogen-Activated Protein Kinase 4 Is Obligatory for Late Pollen and Early Fruit Development in Tomato. Hortic. Res..

[188] Secgin Z., Uluisik S., Yıldırım K., Abdulla M.F., Mostafa K., Kavas M. (2022). Genome-Wide Identification of the Aconitase Gene Family in Tomato (*Solanum lycopersicum*) and CRISPR-Based Functional Characterization of SlACO2 on Male-Sterility. Int. J. Mol. Sci..

[189] Dai X., Han H., Huang W., Zhao L., Song M., Cao X., Liu C., Niu X., Lang Z., Ma C. (2022). Generating Novel Male Sterile Tomatoes by Editing Respiratory Burst Oxidase Homolog Genes. Front. Plant Sci..

[190] Wang Z.-H., Liu S., Zhang Q., Jiang J. (2021). RNA Interference Silencing of the Cytoplasmic Invertases SlCIN7 Leads to Reduction in Pollen Viability and Parthenocarpic Fruit in Tomato. Gene.

[191] Xie D.-L., Huang H.-M., Zhou C.-Y., Liu C.-X., Kanwar M.K., Qi Z.-Y., Zhou J. (2022). HsfA1a Confers Pollen Thermotolerance through Upregulating Antioxidant Capacity, Protein Repair, and Degradation in *Solanum lycopersicum* L.. Hortic. Res..

[192] Pérez-Martín F., Yuste-Lisbona F.J., Pineda B., García-Sogo B., del Olmo I., de Dios Alché J., Egea I., Flores F.B., Piñeiro M., Jarillo J.A. (2018). Developmental Role of the Tomato Mediator Complex Subunit MED18 in Pollen Ontogeny. Plant J..

[193] Wang Y., Hu Z., Zhang J., Yu X., Guo J.-E., Liang H., Liao C., Chen G. (2018). Silencing SlMED18, Tomato Mediator Subunit 18 Gene, Restricts Internode Elongation and Leaf Expansion. Sci. Rep..

[194] Gan Z., Feng Y., Wu T., Wang Y., Xu X., Zhang X., Han Z. (2019). Downregulation of the Auxin Transporter Gene SlPIN8 Results in Pollen Abortion in Tomato. Plant Mol. Biol..

[195] Dai S., Kai W., Liang B., Wang J., Jiang L., Du Y., Sun Y., Leng P. (2018). The Functional Analysis of SlNCED1 in Tomato Pollen Development. Cell. Mol. Life Sci..

[196] Deslous P., Bournonville C., Decros G., Okabe Y., Mauxion J.-P., Jorly J., Gadin S., Brès C., Mori K., Ferrand C. (2021). Overproduction of Ascorbic Acid Impairs Pollen Fertility in Tomato. J. Exp. Bot..

[197] Jansma S.Y., Sergeeva L.I., Tikunov Y.M., Kohlen W., Ligterink W., Rieu I. (2022). Low Salicylic Acid Level Improves Pollen Development under Long-Term Mild Heat Conditions in Tomato. Front. Plant Sci..

[198] Althiab-Almasaud R., Chen Y., Maza E., Djari A., Frasse P., Mollet J.-C., Mazars C., Jamet E., Chervin C. (2021). Ethylene Signaling Modulates Tomato Pollen Tube Growth through Modifications of Cell Wall Remodeling and Calcium Gradient. Plant J..

[199] Yan M.-Y., Xie D.-L., Cao J.-J., Xia X.-J., Shi K., Zhou Y.-H., Zhou J., Foyer C.H., Yu J.-Q. (2020). Brassinosteroid-mediated Reactive Oxygen Species Are Essential for Tapetum Degradation and Pollen Fertility in Tomato. Plant J..

[200] Niwa T., Suzuki T., Takebayashi Y., Ishiguro R., Higashiyama T., Sakakibara H., Ishiguro S. (2018). Jasmonic Acid Facilitates Flower Opening and Floral Organ Development through the Upregulated Expression of SlMYB21 Transcription Factor in Tomato. Biosci. Biotechnol. Biochem..

[201] Julius B.T., Leach K.A., Tran T.M., Mertz R.A., Braun D.M. (2017). Sugar Transporters in Plants: New Insights and Discoveries. Plant Cell Physiol..

[202] Ko H.-Y., Tseng H.-W., Ho L.-H., Wang L., Chang T.-F., Lin A., Ruan Y.-L., Neuhaus H.E., Guo W.-J. (2022). Hexose Translocation Mediated by *Sl*SWEET5b Is Required for Pollen Maturation in *Solanum lycopersicum*. Plant Physiol..

[203] Cai Y., Yin L., Tu W., Deng Z., Yan J., Dong W., Gao H., Xu J., Zhang N., Wang J. (2021). Ectopic Expression of VvSUC27 Induces Stenospermocarpy and Sugar Accumulation in Tomato Fruits. Front. Plant Sci..

[204] Liu H.-K., Li Y.-J., Wang S.-J., Yuan T.-L., Huang W.-J., Dong X., Pei J.-Q., Zhang D., McCormick S., Tang W.-H. (2020). Kinase Partner Protein Plays a Key Role in Controlling the Speed and Shape of Pollen Tube Growth in Tomato. Plant Physiol..

[205] Salazar-Sarasua B., López-Martín M.J., Roque E., Hamza R., Cañas L.A., Beltrán J.P., Gómez-Mena C. (2022). The Tapetal Tissue Is Essential for the Maintenance of Redox Homeostasis during Microgametogenesis in Tomato. Plant J..

[206] Micol-Ponce R., García-Alcázar M., Lebrón R., Capel C., Pineda B., García-Sogo B., Alché J.D.D., Ortiz-Atienza A., Bretones S., Yuste-Lisbona F.J. (2023). Tomato *POLLEN DEFICIENT 2* Encodes a G-Type Lectin Receptor Kinase Required for Viable Pollen Grain Formation. J. Exp. Bot..

[207] Pan C., Yang D., Zhao X., Liu Y., Li M., Ye L., Ali M., Yu F., Lamin-Samu A.T., Fei Z. (2021). PIF4 Negatively Modulates Cold Tolerance in Tomato Anthers via Temperature-Dependent Regulation of Tapetal Cell Death. Plant Cell.

[208] Yang D., Liu Y., Ali M., Ye L., Pan C., Li M., Zhao X., Yu F., Zhao X., Lu G. (2022). Phytochrome Interacting Factor 3 Regulates Pollen Mitotic Division through Auxin Signalling and Sugar Metabolism Pathways in Tomato. New Phytol..

[209] Kravchik M., Stav R., Belausov E., Arazi T. (2019). Functional Characterization of MicroRNA171 Family in Tomato. Plants.

[210] Huang W., Peng S., Xian Z., Lin D., Hu G., Yang L., Ren M., Li Z. (2017). Overexpression of a Tomato MiR171 Target Gene *SlGRAS24* Impacts Multiple Agronomical Traits via Regulating Gibberellin and Auxin Homeostasis. Plant Biotechnol. J..

[211] Keller M., Schleiff E., Simm S. (2020). MiRNAs Involved in Transcriptome Remodeling during Pollen Development and Heat Stress Response in *Solanum lycopersicum*. Sci. Rep..

[212] Guo X., Zhao J., Chen Z., Qiao J., Zhang Y., Shen H., Hu Z. (2022). CRISPR/Cas9-Targeted Mutagenesis of *SlCMT4* Causes Changes in Plant Architecture and Reproductive Organs in Tomato. Hortic. Res..

[213] Rick C.M. (1988). Tomato-like Nightshades: Affinities, Autoecology, and Breeders’ Opportunities. Econ. Bot..

[214] Cheng M.-Z., Gong C., Zhang B., Qu W., Qi H.-N., Chen X.-L., Wang X.-Y., Zhang Y., Liu J.-Y., Ding X.-D. (2021). Morphological and Anatomical Characteristics of Exserted Stigma Sterility and the Location and Function of SlLst (*Solanum lycopersicum* Long Styles) Gene in Tomato. Züchter Genet. Breed. Res..

[215] Shang L., Song J., Yu H., Wang X., Yu C., Wang Y., Li F., Lu Y., Wang T., Ouyang B. (2021). A Mutation in a C2H2-Type Zinc Finger Transcription Factor Contributed to the Transition toward Self-Pollination in Cultivated Tomato. Plant Cell.

[216] Dumas C., Knox R.B. (1983). Callose and Determination of Pistil Viability and Incompatibility. Züchter Genet. Breed. Res..

[217] Nal M., Vardar F., Ayturk Z. (2013). Callose in Plant Sexual Reproduction. Current Progress in Biological Research.

[218] Pei Y., Xue Q., Zhang Z., Shu P., Deng H., Bouzayen M., Hong Y., Liu M. (2023). *β-1,3-GLUCANASE10* Regulates Tomato Development and Disease Resistance by Modulating Callose Deposition. Plant Physiol..

[219] Sarma S., Pandey A.K., Sharma K., Ravi M., Sreelakshmi Y., Sharma R. (2018). MutS-Homolog2 Silencing Generates Tetraploid Meiocytes in Tomato (*Solanum lycopersicum*). Plant Direct.

[220] Strelnikova S.R., Krinitsina A.A., Komakhin R.A. (2021). Effective RNAi-Mediated Silencing of the Mismatch Repair MSH2 Gene Induces Sterility of Tomato Plants but Not an Increase in Meiotic Recombination. Genes.

[221] Qin X., Li W., Liu Y., Tan M., Ganal M., Chetelat R.T. (2018). A Farnesyl Pyrophosphate Synthase Gene Expressed in Pollen Functions in *S*-RNase-independent Unilateral Incompatibility. Plant J..

[222] Muñoz-Sanz J.V., Tovar-Méndez A., Lu L., Dai R., McClure B. (2021). A Cysteine-Rich Protein, SpDIR1L, Implicated in S-RNase-Independent Pollen Rejection in the Tomato (*Solanum* Section *Lycopersicon*) Clade. Int. J. Mol. Sci..

[223] Tran L.T., Sugimoto K., Kasozi M., Mitalo O.W., Ezura H. (2023). Pollination, Pollen Tube Growth, and Fertilization Independently Contribute to Fruit Set and Development in Tomato. Front. Plant Sci..

[224] Lombardo F., Gramazio P., Ezura H. (2021). Increase in Phloem Area in the Tomato Hawaiian Skirt Mutant Is Associated with Enhanced Sugar Transport. Genes.

[225] Kusano M., Worarad K., Fukushima A., Kamiya K., Mitani Y., Okazaki Y., Higashi Y., Nakabayashi R., Kobayashi M., Mori T. (2022). Transcriptomic, Hormonomic and Metabolomic Analyses Highlighted the Common Modules Related to Photosynthesis, Sugar Metabolism and Cell Division in Parthenocarpic Tomato Fruits during Early Fruit Set. Cells.

[226] Gorguet B., van Heusden A.W., Lindhout P. (2005). Parthenocarpic Fruit Development in Tomato. Plant Biol..

[227] de Jong M., Mariani C., Vriezen W.H. (2009). The Role of Auxin and Gibberellin in Tomato Fruit Set. J. Exp. Bot..

[228] Serrani J.C., Fos M., Atarés A., García-Martínez J.L. (2007). Effect of Gibberellin and Auxin on Parthenocarpic Fruit Growth Induction in the Cv Micro-Tom of Tomato. J. Plant Growth Regul..

[229] Srivastava A., Handa A.K. (2005). Hormonal Regulation of Tomato Fruit Development: A Molecular Perspective. J. Plant Growth Regul..

[230] Hu J., Israeli A., Ori N., Sun T.-P. (2018). The Interaction between DELLA and ARF/IAA Mediates Crosstalk between Gibberellin and Auxin Signaling to Control Fruit Initiation in Tomato. Plant Cell.

[231] Wang H., Jones B., Li Z., Frasse P., Delalande C., Regad F., Chaabouni S., Latché A., Pech J.-C., Bouzayen M. (2005). The Tomato *Aux*/*IAA* Transcription Factor *IAA9* Is Involved in Fruit Development and Leaf Morphogenesis. Plant Cell.

[232] He M., Song S., Zhu X., Lin Y., Pan Z., Chen L., Chen D., Hu G., Huang B., Chen M. (2021). SlTPL1 Silencing Induces Facultative Parthenocarpy in Tomato. Front. Plant Sci..

[233] Kim J.-S., Ezura K., Lee J., Ariizumi T., Ezura H. (2019). Genetic Engineering of Parthenocarpic Tomato Plants Using Transient SlIAA9 Knockdown by Novel Tissue-Specific Promoters. Sci. Rep..

[234] Ueta R., Abe C., Watanabe T., Sugano S.S., Ishihara R., Ezura H., Osakabe Y., Osakabe K. (2017). Rapid Breeding of Parthenocarpic Tomato Plants Using CRISPR/Cas9. Sci. Rep..

[235] Tomlinson L., Yang Y., Emenecker R., Smoker M., Taylor J., Perkins S., Smith J., MacLean D., Olszewski N.E., Jones J.D.G. (2019). Using CRISPR/Cas9 Genome Editing in Tomato to Create a Gibberellin-responsive Dominant Dwarf DELLA Allele. Plant Biotechnol. J..

[236] Shinozaki Y., Ezura K., Hu J., Okabe Y., Bénard C., Prodhomme D., Gibon Y., Sun T.-P., Ezura H., Ariizumi T. (2018). Identification and Functional Study of a Mild Allele of SlDELLA Gene Conferring the Potential for Improved Yield in Tomato. Sci. Rep..

[237] Illouz-Eliaz N., Ramon U., Shohat H., Blum S., Livne S., Mendelson D., Weiss D. (2019). Multiple Gibberellin Receptors Contribute to Phenotypic Stability under Changing Environments. Plant Cell.

[238] Illouz-Eliaz N., Nissan I., Nir I., Ramon U., Shohat H., Weiss D. (2020). Mutations in the Tomato Gibberellin Receptors Suppress Xylem Proliferation and Reduce Water Loss under Water-Deficit Conditions. J. Exp. Bot..

[239] María Victoria B., Claudia B., Cecilia D., Mauricio H.-C., Silvana B.B., Estela M.V., Eduardo Z. (2003). MADS-box genes expressed during tomato seed and fruit development. Plant Mol. Biol..

[240] Pnueli L., Hareven D., Rounsley S.D., Yanofsky M.F., Lifschitz E. (1994). Isolation of the Tomato AGAMOUS Gene TAG1 and Analysis of Its Homeotic Role in Transgenic Plants. Plant Cell.

[241] Vrebalov J., Pan I.L., Arroyo A.J.M., McQuinn R., Chung M., Poole M., Rose J., Seymour G., Grandillo S., Giovannoni J. (2009). Fleshy Fruit Expansion and Ripening Are Regulated by the Tomato *SHATTERPROOF* Gene *TAGL1*. Plant Cell.

[242] Ribelles C., García-Sogo B., Yuste-Lisbona F.J., Atarés A., Castañeda L., Capel C., Lozano R., Moreno V., Pineda B. (2019). Alq Mutation Increases Fruit Set Rate and Allows the Maintenance of Fruit Yield under Moderate Saline Conditions. J. Exp. Bot..

[243] Klap C., Yeshayahou E., Bolger A.M., Arazi T., Gupta S.K., Shabtai S., Usadel B., Salts Y., Barg R. (2017). Tomato Facultative Parthenocarpy Results from Sl*AGAMOUS-LIKE 6* Loss of Function. Plant Biotechnol. J..

[244] Gupta S.K., Barg R., Arazi T. (2021). Tomato *Agamous-Like6* Parthenocarpy Is Facilitated by Ovule Integument Reprogramming Involving the Growth Regulator *KLUH*. Plant Physiol..

[245] Huang B., Routaboul J.-M., Liu M., Deng W., Maza E., Mila I., Hu G., Zouine M., Frasse P., Vrebalov J.T. (2017). Overexpression of the Class D MADS-Box Gene Sl-AGL11 Impacts Fleshy Tissue Differentiation and Structure in Tomato Fruits. J. Exp. Bot..

[246] Rojas-Gracia P., Roque E., Medina M., Rochina M., Hamza R., Angarita-Díaz M.P., Moreno V., Pérez-Martín F., Lozano R., Cañas L. (2017). The Parthenocarpic *Hydra* Mutant Reveals a New Function for a *SPOROCYTELESS*-like Gene in the Control of Fruit Set in Tomato. New Phytol..

[247] Hao S., Ariizumi T., Ezura H. (2017). *SEXUAL STERILITY* Is Essential for Both Male and Female Gametogenesis in Tomato. Plant Cell Physiol..

[248] da Silva E.M., Silva G.F.F.E., Bidoia D.B., da Silva Azevedo M., de Jesus F.A., Pino L.E., Peres L.E.P., Carrera E., López-Díaz I., Nogueira F.T.S. (2017). MicroRNA159-targeted *SlGAMYB* Transcription Factors Are Required for Fruit Set in Tomato. Plant J..

[249] Zhao P., Wang F., Deng Y., Zhong F., Tian P., Lin D., Deng J., Zhang Y., Huang T. (2022). Sly-miR159 Regulates Fruit Morphology by Modulating GA Biosynthesis in Tomato. Plant Biotechnol. J..

[250] Takei H., Shinozaki Y., Yano R., Kashojiya S., Hernould M., Chevalier C., Ezura H., Ariizumi T. (2019). Loss-of-Function of a Tomato Receptor-like Kinase Impairs Male Fertility and Induces Parthenocarpic Fruit Set. Front. Plant Sci..

[251] Schubert R., Dobritzsch S., Gruber C., Hause G., Athmer B., Schreiber T., Marillonnet S., Okabe Y., Ezura H., Acosta I.F. (2019). Tomato MYB21 Acts in Ovules to Mediate Jasmonate-Regulated Fertility. Plant Cell.

[252] Xu J., Li J., Cui L., Zhang T., Wu Z., Zhu P.-Y., Meng Y.-J., Zhang K.-J., Yu X.-Q., Lou Q.-F. (2017). New Insights into the Roles of Cucumber TIR1 Homologs and miR393 in Regulating Fruit/Seed Set Development and Leaf Morphogenesis. BMC Plant Biol..

[253] Liu S., Zhang Y., Feng Q., Qin L., Pan C., Lamin-Samu A.T., Lu G. (2018). Tomato AUXIN RESPONSE FACTOR 5 Regulates Fruit Set and Development via the Mediation of Auxin and Gibberellin Signaling. Sci. Rep..

[254] Yang Y., Tang K., Datsenka T.U., Liu W., Lv S., Lang Z., Wang X., Gao J., Wang W., Nie W. (2019). Critical Function of DNA Methyltransferase 1 in Tomato Development and Regulation of the DNA Methylome and Transcriptome. J. Integr. Plant Biol..

[255] Nambeesan S.U., Mattoo A.K., Handa A.K. (2019). Nexus between Spermidine and Floral Organ Identity and Fruit/Seed Set in Tomato. Front. Plant Sci..

[256] Okabe Y., Yamaoka T., Ariizumi T., Ushijima K., Kojima M., Takebayashi Y., Sakakibara H., Kusano M., Shinozaki Y., Pulungan S.I. (2019). Aberrant Stamen Development Is Associated with Parthenocarpic Fruit Set through Up-Regulation of Gibberellin Biosynthesis in Tomato. Plant Cell Physiol..

[257] Wang H., Wu T., Liu J., Cong L., Zhu Y., Zhai R., Yang C., Wang Z., Ma F., Xu L. (2020). PbGA20ox2 Regulates Fruit Set and Induces Parthenocarpy by Enhancing GA4 Content. Front. Plant Sci..

[258] Matsuo S., Miyatake K., Endo M., Urashimo S., Kawanishi T., Negoro S., Shimakoshi S., Fukuoka H. (2020). Loss of Function of the *Pad-1* Aminotransferase Gene, Which Is Involved in Auxin Homeostasis, Induces Parthenocarpy in Solanaceae Plants. Proc. Natl. Acad. Sci. USA.

[259] Shinozaki Y., Beauvoit B.P., Takahara M., Hao S., Ezura K., Andrieu M.-H., Nishida K., Mori K., Suzuki Y., Kuhara S. (2020). Fruit Setting Rewires Central Metabolism via Gibberellin Cascades. Proc. Natl. Acad. Sci. USA.

[260] Abe-Hara C., Yamada K., Wada N., Ueta R., Hashimoto R., Osakabe K., Osakabe Y. (2021). Effects of the Sliaa9 Mutation on Shoot Elongation Growth of Tomato Cultivars. Front. Plant Sci..

[261] Nagata T., Lombardo F., Ezura H. (2021). Complementation of the Tomato *HWS* Gene with Its Arabidopsis Counterpart Demonstrates Conservation of the Gene Function between Both Species. Plant Biotechnol..

[262] Clepet C., Devani R.S., Boumlik R., Hao Y., Morin H., Marcel F., Verdenaud M., Mania B., Brisou G., Citerne S. (2021). The miR166–SlHB15A Regulatory Module Controls Ovule Development and Parthenocarpic Fruit Set under Adverse Temperatures in Tomato. Mol. Plant.

[263] Zhang H., Han W., Wang H., Cong L., Zhai R., Yang C., Wang Z., Xu L. (2021). Downstream of GA4, PbCYP78A6 Participates in Regulating Cell Cycle-Related Genes and Parthenogenesis in Pear (*Pyrus bretshneideri* Rehd.). BMC Plant Biol..

[264] Kashojiya S., Lu Y., Takayama M., Komatsu H., Minh L.H.T., Nishida K., Shirasawa K., Miura K., Nonaka S., Masuda J.-I. (2022). Modification of Tomato Breeding Traits and Plant Hormone Signaling by Target-AID, the Genome-Editing System Inducing Efficient Nucleotide Substitution. Hortic. Res..

[265] Hu J., Li X., Sun T.-P. (2023). Four Class A AUXIN RESPONSE FACTORs Promote Tomato Fruit Growth despite Suppressing Fruit Set. Nat. Plants.

[266] Ding J., Chen B., Xia X., Mao W., Shi K., Zhou Y., Yu J. (2013). Cytokinin-Induced Parthenocarpic Fruit Development in Tomato Is Partly Dependent on Enhanced Gibberellin and Auxin Biosynthesis. PLoS ONE.

[267] Vriezen W.H., Feron R., Maretto F., Keijman J., Mariani C. (2008). Changes in Tomato Ovary Transcriptome Demonstrate Complex Hormonal Regulation of Fruit Set. New Phytol..

[268] Nitsch L.M.C., Oplaat C., Feron R., Ma Q., Wolters-Arts M., Hedden P., Mariani C., Vriezen W.H. (2009). Abscisic Acid Levels in Tomato Ovaries Are Regulated by LeNCED1 and SlCYP707A1. Planta.

[269] Shinozaki Y., Hao S., Kojima M., Sakakibara H., Ozeki-Iida Y., Zheng Y., Fei Z., Zhong S., Giovannoni J.J., Rose J.K.C. (2015). Ethylene Suppresses Tomato (*Solanum lycopersicum*) Fruit Set through Modification of Gibberellin Metabolism. Plant J..

[270] Bishop G.J., Nomura T., Yokota T., Harrison K., Noguchi T., Fujioka S., Takatsuto S., Jones J.D.G., Kamiya Y. (1999). The Tomato DWARF Enzyme Catalyses C-6 Oxidation in Brassinosteroid Biosynthesis. Proc. Natl. Acad. Sci. USA.

[271] Cebrián G., Segura M., Martínez J., Iglesias-Moya J., Martínez C., Garrido D., Jamilena M. (2023). Jasmonate-Deficient Mutant *Lox3a* Reveals Crosstalk between Jasmonate and Ethylene in the Differential Regulation of Male and Female Flower Opening and Early Fruit Development in *Cucurbita Pepo*. J. Exp. Bot..

[272] Khan M., Luo B., Hu M., Fu S., Liu J., Jiang M., Zhao Y., Huang S., Wang S., Wang X. (2022). Brassinosteroid Signaling Downstream Suppressor BIN2 Interacts with SLFRIGIDA-LIKE to Induce Early Flowering in Tomato. Int. J. Mol. Sci..

[273] Hong J., Lee H., Lee J., Kim H., Ryu H. (2019). ABSCISIC ACID-INSENSITIVE 3 Is Involved in Brassinosteroid-Mediated Regulation of Flowering in Plants. Plant Physiol. Biochem..

[274] Hu G., Huang B., Wang K., Frasse P., Maza E., Djari A., Benhamed M., Gallusci P., Li Z., Zouine M. (2021). Histone Posttranslational Modifications Rather than DNA Methylation Underlie Gene Reprogramming in Pollination-dependent and Pollination-independent Fruit Set in Tomato. New Phytol..

[275] Hawar A., Xiong S., Yang Z., Sun B. (2022). Histone Acetyltransferase SlGCN5 Regulates Shoot Meristem and Flower Development in *Solanum lycopersicum*. Front. Plant Sci..

[276] Molesini B., Pennisi F., Vitulo N., Pandolfini T. (2023). MicroRNAs Associated with AGL6 and IAA9 Function in Tomato Fruit Set. BMC Res. Notes.

[277] Yang Z., Yang C., Wang Z., Yang Z., Chen D., Wu Y. (2019). LncRNA Expression Profile and ceRNA Analysis in Tomato during Flowering. PLoS ONE.

[278] Yang Z., Yang Z., Yang C., Wang Z., Chen D., Xie Y., Wu Y. (2020). Identification and Genetic Analysis of Alternative Splicing of Long Non-Coding RNAs in Tomato Initial Flowering Stage. Genomics.

[279] Mubarok S., Jadid N., Widiastuti A., Derajat Matra D., Budiarto R., Lestari F.W., Nuraini A., Suminar E., Pradana Nur Rahmat B., Ezura H. (2023). Parthenocarpic Tomato Mutants, Iaa9-3 and Iaa9-5, Show Plant Adaptability and Fruiting Ability under Heat-Stress Conditions. Front. Plant Sci..

[280] Chong L., Xu R., Huang P., Guo P., Zhu M., Du H., Sun X., Ku L., Zhu J.-K., Zhu Y. (2022). The Tomato OST1–VOZ1 Module Regulates Drought-Mediated Flowering. Plant Cell.

[281] Yuan S., Kawasaki S., Abdellatif I.M.Y., Nishida K., Kondo A., Ariizumi T., Ezura H., Miura K. (2021). Efficient Base Editing in Tomato Using a Highly Expressed Transient System. Plant Cell Rep..

[282] Hunziker J., Nishida K., Kondo A., Kishimoto S., Ariizumi T., Ezura H. (2020). Multiple Gene Substitution by Target-AID Base-Editing Technology in Tomato. Sci. Rep..

[283] Lu Y., Tian Y., Shen R., Yao Q., Zhong D., Zhang X., Zhu J.-K. (2021). Precise Genome Modification in Tomato Using an Improved Prime Editing System. Plant Biotechnol. J..

[284] Van Vu T., Nguyen N.T., Kim J., Das S., Lee J., Kim J.-Y. (2022). The Obstacles and Potential Solution Clues of Prime Editing Applications in Tomato. Biodes. Res..

[285] Kwon C.-T., Heo J., Lemmon Z.H., Capua Y., Hutton S.F., Van Eck J., Park S.J., Lippman Z.B. (2020). Rapid Customization of Solanaceae Fruit Crops for Urban Agriculture. Nat. Biotechnol..

[286] Rodríguez-Leal D., Lemmon Z.H., Man J., Bartlett M.E., Lippman Z.B. (2017). Engineering Quantitative Trait Variation for Crop Improvement by Genome Editing. Cell.

[287] Li T., Yang X., Yu Y., Si X., Zhai X., Zhang H., Dong W., Gao C., Xu C. (2018). Domestication of Wild Tomato Is Accelerated by Genome Editing. Nat. Biotechnol..

[288] Zsögön A., Čermák T., Naves E.R., Notini M.M., Edel K.H., Weinl S., Freschi L., Voytas D.F., Kudla J., Peres L.E.P. (2018). De Novo Domestication of Wild Tomato Using Genome Editing. Nat. Biotechnol..

[289] Sierra-Orozco E., Shekasteband R., Illa-Berenguer E., Snouffer A., van der Knaap E., Lee T.G., Hutton S.F. (2021). Identification and Characterization of GLOBE, a Major Gene Controlling Fruit Shape and Impacting Fruit Size and Marketability in Tomato. Hortic. Res..

[290] Lin W., Gupta S.K., Arazi T., Spitzer-Rimon B. (2021). MIR172d Is Required for Floral Organ Identity and Number in Tomato. Int. J. Mol. Sci..

[291] Tsatsakis A.M., Nawaz M.A., Kouretas D., Balias G., Savolainen K., Tutelyan V.A., Golokhvast K.S., Lee J.D., Yang S.H., Chung G. (2017). Environmental Impacts of Genetically Modified Plants: A Review. Environ. Res..

[292] Timerbaev V., Pushin A., Dolgov S. (2019). Production of Marker-Free Tomato Plants Expressing the Supersweet Protein Thaumatin II Gene under the Control of Predominantly Fruit-Specific Promoters. Plant Cell Tissue Organ Cult..

[293] Ahmad A., Jamil A., Munawar N. (2023). GMOs or Non-GMOs? The CRISPR Conundrum. Front. Plant Sci..

